# The Role of Cell Volume in the Dynamics of Seizure, Spreading Depression, and Anoxic Depolarization

**DOI:** 10.1371/journal.pcbi.1004414

**Published:** 2015-08-14

**Authors:** Ghanim Ullah, Yina Wei, Markus A Dahlem, Martin Wechselberger, Steven J Schiff

**Affiliations:** 1 Department of Physics, University of South Florida, Tampa, Florida 33620, United States of America; 2 Department of Cell Biology and Neuroscience, University of California, Riverside, California 92501 United States of America; 3 Humboldt-Universität zu Berlin, 10115 Berlin, Germany; 4 School of Mathematics and Statistics, University of Sydney, New South Wales, 2006, Australia; 5 Center for Neural Engineering, Departments of Engineering Science and Mechanics, Neurosurgery, and Physics, The Pennsylvania State University, University Park, Pennsylvania 16802, United States of America; Université Paris Descartes, Centre National de la Recherche Scientifique, FRANCE

## Abstract

Cell volume changes are ubiquitous in normal and pathological activity of the brain. Nevertheless, we know little of how cell volume affects neuronal dynamics. We here performed the first detailed study of the effects of cell volume on neuronal dynamics. By incorporating cell swelling together with dynamic ion concentrations and oxygen supply into Hodgkin-Huxley type spiking dynamics, we demonstrate the spontaneous transition between epileptic seizure and spreading depression states as the cell swells and contracts in response to changes in osmotic pressure. Our use of volume as an order parameter further revealed a dynamical definition for the experimentally described physiological ceiling that separates seizure from spreading depression, as well as predicted a second ceiling that demarcates spreading depression from anoxic depolarization. Our model highlights the neuroprotective role of glial K buffering against seizures and spreading depression, and provides novel insights into anoxic depolarization and the relevant cell swelling during ischemia. We argue that the dynamics of seizures, spreading depression, and anoxic depolarization lie along a continuum of the repertoire of the neuron membrane that can be understood only when the dynamic ion concentrations, oxygen homeostasis,and cell swelling in response to osmotic pressure are taken into consideration. Our results demonstrate the feasibility of a unified framework for a wide range of neuronal behaviors that may be of substantial importance in the understanding of and potentially developing universal intervention strategies for these pathological states.

## Author Summary

Massive rearrangement of ions across the plasma membrane and changes in cellular volume are common features of states such as seizures, spreading depression, and ischemia. In this paper, we focus on how volume itself influences neuronal activity. We build a unified computational framework for a wide range of neuronal behaviors by exploiting their previously unexplored common features. By combining the dynamic ion concentrations and volume, conservation of charge, and the energy requirements of the cell within a Hodgkin-Huxley type framework, we demonstrate the feasibility of a comprehensive framework encompassing a wide range of neuronal behaviors. We show the spontaneous transition of a neuron between seizure and spreading depression when the cell swells and contracts in response to varying osmotic pressure as a result of the rearrangement of different ions. Our model closely reproduces anoxic depolarization and relevant neuronal swelling during ischemia and reveals a dynamical definition for the experimentally described physiological ceilings that demarcate seizure from spreading depression and spreading depression from anoxic depolarization. This study opens up a new way of studying neuronal behavior where different states need not be treated separately but rather as a dynamical continuum of the neuronal membrane potential and its microenvironment.

## Introduction

Cells swell during a wide variety of pathologies, including trauma, ischemia, hypoxia, seizures, and spreading depression [1–3]. Changes in osmolality can change the susceptibility to epileptiform activity [4–6], and affect the amplitude of intra- and extracellularly recorded electrical signals [7]. Cells also change their volume during normal activity, and the change in cell size during individual action potentials has been estimated [8, 9]. Despite this ubiquity of observed phenomena, the effect of cell swelling on single cell behavior is incompletely understood.

It is now accepted that the dynamic microenvironment within the extracellular space (ECS), modified by ionic fluxes from neurons, glia, and blood vessels, plays a critical role in neuronal behavior [1]. In particular, pathological states involving excessive neuronal depolarization such as epileptic seizure (SZ), spreading depression (SD), and anoxic depolarization (AD) during ischemia are characterized by major rearrangements of various ions across the cell membrane and neuronal microenvironment [1, 10–16]. In each of these three conditions, collapse of transmembrane ionic gradients requires enhanced oxygen and glucose consumption required by active transport systems to reestablish the gradients [17, 18]. For the purpose of this paper, we define SZ, SD, and AD respectively as the ion concentrations-induced high-frequency bursts not usually seen in the normal condition of the same cell [1, 19], the nearly complete depolarization of the cell’s membrane potential that recovers spontaneously on the scale of seconds [13, 19], and the nearly complete depolarization of the cell’s membrane potential triggered by oxygen (*O*_2_) and glucose deprivation (OGD) that may or may not recover depending on the cell type after *O*_2_ and glucose is restored [1, 20, 21].

During the pathological states mentioned above, the massive rearrangement of ions across plasma membrane drives water molecules from the extra- to intracellular space leading cell swelling. For example, pyramidal neurons in slices from cortical layer V swell by as much as 60% during AD caused by 20 minutes OGD [20]. Although lacking functional aquaporins, neurons swell significantly in response to OGD and extracellular *K* ^+^ elevations [22]. Although still debated, the *K* ^+^/*Cl* ^−^ and *Na* ^+^/*K* ^+^/2*Cl* ^−^ cotransporters are suspected to mediate the entry of water molecules into neurons [23, 24]. Astrocytes on the other hand, express aquaporins [25]. The clearance of excessive *K* ^+^ due to high neuronal activity by astrocytes leads to osmotic gradients resulting in water influx through aquaporins and astrocytic dilation [26–28]. This paper focusses on the role of neuronal swelling in response to osmolality changes in cell behavior without considering the specifics of pathways involved in the water influx.

Despite the fact that SD was first observed by Leão as the silencing of spontaneous electrical activity during experiments on epileptic SZs, the two phenomena have long been considered separate physiological events [1]. They are characterized by different patterns of neuronal activities [29–31], characteristic ionic changes [13–16, 19], and known interactions with oxygen [17, 32]. By expanding the Hodgkin-Huxley type framework to incorporate conservation of particles and charge, and accounting for the energy required to restore ionic gradients, we recently uncovered a unified mechanism for SZ and SD [33]. Specifically, we showed that a wide-range of neuronal behaviors can be accounted for as a function of the cell’s extracellular potassium concentration and oxygen supply. More recently, Hübel and Dahlem performed a detailed bifurcation analysis of different time-scales arising from the consideration of dynamic ion concentrations in conjunction with Hodgkin-Huxley type framework [34].

Extensive work has been done on the role of ion pumps, channels, and transporters in stroke [35]. Recently, Andrew and colleagues showed that neuronal populations in lower brain regions such as the hypothalamus are resistant while those in higher brain regions such as neocortex are more susceptible to ischemic injury [20]. They further showed that the thalamus-hypothalamus interface represents a discrete boundary where neurons in thalamus are more vulnerable than hypothalamus to ischemia, generating stronger AD in response to OGD, and do not recover as readily after restoring normal *O*_2_ and glucose supply [21]. The authors postulated that the variability of ATP-dependent *Na* ^+^ − *K* ^+^ pumps in these regions could lead to the contrasting neuronal response in OGD conditions.

In this paper, we explore the effect of cell swelling on neuronal behavior by demonstrating the ability of cell volume to act as a bifurcation parameter. We seek a better understanding of how human brain cells respond to osmotic pressure-induced swelling in states such as SZ, SD, and AD, and universal intervention strategies for controlling these conditions. Here, we show that spontaneous transition between SZ and SD can be seen in a model neuron if volumetric changes in response to intense neuronal activity and ionic fluxes are taken into account. Without any adjustments, our model behaves in similar fashion as *in vitro* experiments under OGD. We further show that the variability in the geometry and microenvironment of neurons could play a significant part in their differential response in OGD conditions observed in *in vitro* experiments in different brain regions. Based on our results, we conclude that combining ion concentration dynamics during spiking with the sizes of intra- and extracellular spaces supports a unified framework for epileptic SZ, SD, and AD.

## Results

### Transition between SZ and SD states

**Fig 1:**
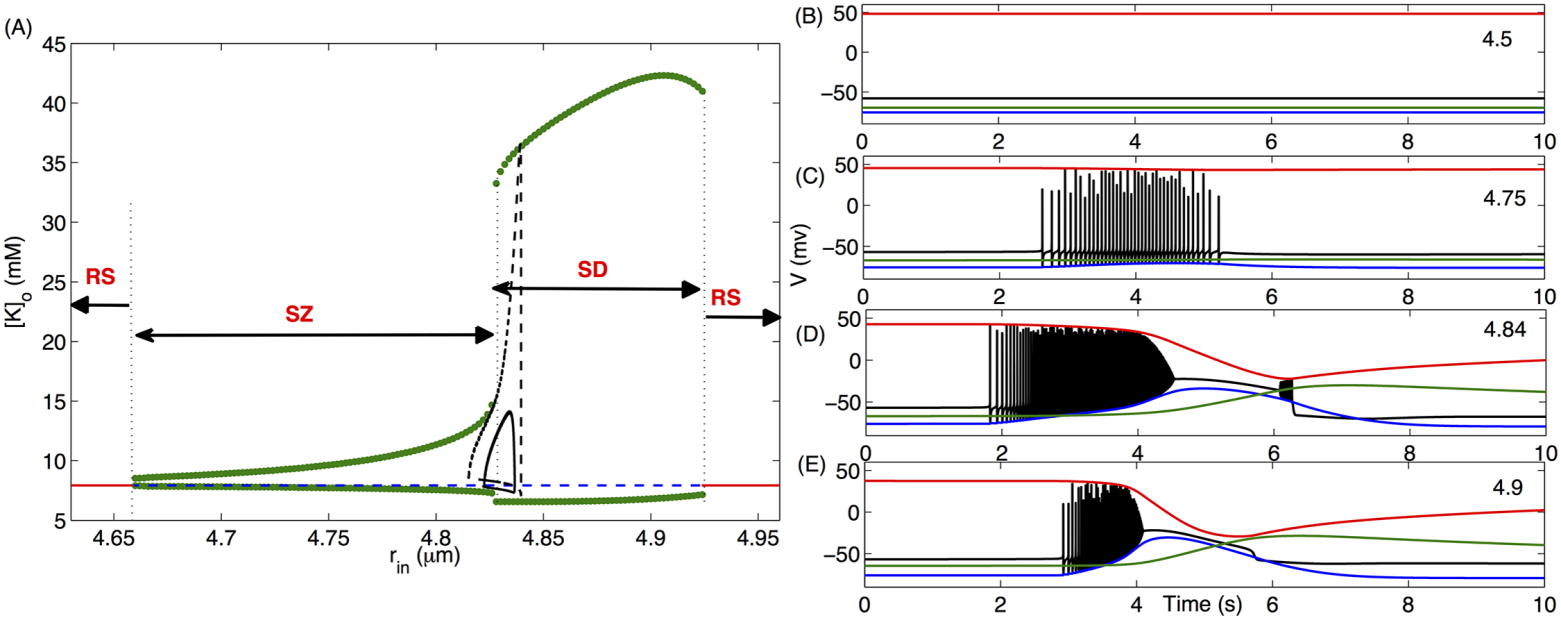
Cell shows a variety of behaviors as we vary its size. Here we consider *Vol* (Eq 14) as a bifurcation parameter and simulate Eqs (1, 3, 5, 7, 10). (A) Bifurcation diagram of [*K*]_*o*_ as a function of *r*_*in*_ where green circles, red solid line, and blue dashed line represent periodic orbit, stable, and unstable steady states respectively. The four regions in the bifurcation diagram marked by SZ, SD, and RS represent the parameter-regions where seizure, spreading depression, and resting states are observed respectively. The black solid and dashed limit cycles represent the change in *r*_*in*_ as different ion concentrations vary during a single SZ at [*K*]_*o*,∞_ = 8mM and 9mM respectively. For the limit cycles, the instantaneous *r*_*in*_ values in the limit cycles are obtained from Eq (13). The four panels on the right show membrane potential (black), reversal potential of *K* ^+^ (blue), *Cl* ^−^ (green), and *Na* ^+^ (red) of the cell with different *r*_*in*_ values given in the right corner of each panel.

We investigate the role of cell size and relative (to intracellular volume) extracellular space on neuronal behavior by varying the radius of the cell, *r*_*in*_, keeping the total radius of cell and extracellular space, *r*_*tot*_, fixed. Thus changing *r*_*in*_ is largely equivalent to changing the ratio of intra- to extracellular volume. Since we are interested in the pathological states of the cell, we use *K* ^+^ concentration in the distant reservoir [*K*]_*o*,∞_ = 8*mM*—a value typically used for inducing SZ in *in vitro* [30]. The behavior of the cell changes dramatically as we increase *r*_*in*_. A bifurcation diagram showing the maximum and minimum of [*K*]_*o*_ as a function of *r*_*in*_ is shown in Fig 1A. Briefly, we fixed *r*_*in*_ and ran the simulation generating a time-trace representing [*K*]_*o*_ versus time. Depending on the *r*_*in*_ value, [*K*]_*o*_ either oscillates or converge to a steady state value. The initial few hundred seconds of the time-trace were discarded as a transient period and the maximum and minimum of [*K*]_*o*_ values in the remaining trace were recorded. The lower and upper markers (green) respectively at a given *r*_*in*_ in Fig 1A represent the maxima and minima of [*K*]_*o*_ oscillations for that particular value of *r*_*in*_. This process was repeated many times, each time incrementing *r*_*in*_ by a small amount. For *r*_*in*_ values, where [*K*]_*o*_ does not oscillate (i.e. [*K*]_*o*_ converges to a steady state), the maxima and minima have the same value and is represented by a line (stable, red; unstable, blue). To capture both stable and unstable behaviors, the steady states were simulated in XPPAUT.

For all *r*_*in*_ < 4.66*μm*, [*K*]_*o*_ remains unchanged and the cell remains in steady state with *V* ≈ −60*mV* (Fig 1B). As we increase *r*_*in*_ above 4.66*μm*, [*K*]_*o*_ enters a periodic orbit via a Hopf bifurcation and starts oscillating with a small amplitude and the cell exhibits spontaneous periodic SZs (Fig 1C) similar to those observed in experiments [36]. There is a sudden increase in the amplitude of [*K*]_*o*_ oscillations at *r*_*in*_ = 4.826*μm* where the peak [*K*]_*o*_ goes well over 26mM—a concentration that is often used for inducing SD [37]. The periodic SZs transform to a behavior where the cell is locked into a depolarized state after burst-spiking and exhibits a few small-amplitude spikes on its way out of the depolarized state (Fig 1D). As we increase *r*_*in*_ further, this state disappears making way for mixed SZ and SD behavior where the high-frequency spiking is followed by the locking of *V* into a depolarized state and the cell comes out of the depolarized state without spiking (Fig 1E). Such mixed states are typically seen in the cells in hypoxic SD [32] or immature physiological conditions [38]. It is worth mentioning that this locking of neuronal membrane into depolarized state is the condition for SD at the single cell level [39]. At the network or tissue level the depolarization may also propagate [40]. This unification of SZ and SD dynamics is supported by the increasing discovery of monogenic mutations in humans that lead to both SZs and migraines [41]. The cell exhibits SZ-SD mixed behavior until it makes a transition to a silent state via another Hopf bifurcation at *r*_*in*_ > 4.924*μm* where *V* remains fixed at a stable resting value.

The bifurcation diagrams for [*Na*]_*i*_, [*K*]_*i*_, and [*Cl*]_*i*_ (similar to Fig 1A) (not shown) show that their behavior contrasts with [*K*]_*o*_. That is, the amplitude (defined as the difference between the maximum and minimum) of [*Na*]_*i*_, [*K*]_*i*_, and [*Cl*]_*i*_ oscillations decreases as we increase *r*_*in*_. At *r*_*in*_ > 4.924*μm*, the relatively larger intracellular volume dominates the extracellular space and these 3 concentrations drop to resting values leading to a return to resting membrane potential. That is, the extremely large intracellular volume leads to low intracellular concentrations that overshadow the effect of the changes in extracellular ion concentrations. The bifurcations in the cell’s behavior described above are qualitatively preserved when [*K*]_*i*_, [*Na*]_*o*_, and [*Cl*]_*o*_ are modeled by rate equations [33] instead of conservation equations (S1 Fig). It is important to point out that the kind of changes in *r*_*in*_ shown in Fig 1A and the rest of the paper are physiologically relevant. For example, the surface area of cortical layer V pyramidal neurons increases by more than 50% in response to OGD [20]. Thus a spherical cell with initial radius of 4.75*μm* would swell to a final radius of 5.81*μm*, shrinking the extracellular space significantly.

The change in the ratio of intra- to extracellular volume as a result of changing *r*_*in*_ plays a major role in the transition between SZ and SD states. Depending on the value of *β*, the cell exhibits steady state, SZ, or SD without any transition between these behaviors as we change *r*_*in*_ if *β* is kept constant (S2(A) Fig). Using *β* as a bifurcation parameter at fixed *r*_*in*_ on the other hand causes the cell to make the transitions between steady state, SZ, and SD (S2(B) Fig). Nevertheless, the cell size per se is an important parameter that together with *β* shapes the bifurcations and the parameter ranges where different behaviors are observed (compare Fig 1A and S2 Fig).

The results in Fig 1 indicate that the size of the cells and how tightly they are packed in the tissue can play a significant role in their dynamics. An important followup question would be: could a cell swell enough so that it would spontaneously go through the transitions shown in Fig 1? To answer this question, we add the volume dynamics given by Eq (14) to our model, where the cell volume depends on the instantaneous ion concentrations. We compute the spontaneous change in *r*_*in*_ as the ion concentrations inside and outside the cell vary during a single SZ (solid black line in Fig 1A). The limit cycle shows that *r*_*in*_ can change enough during one SZ so that the cell would make the transition from SZ to SD regions. A cell with *r*_*in*_ = 4.82*μm* (which is in the SZ region and will exhibit spontaneous SZs similar to Fig 1C) before a SZ starts would swell to a final *r*_*in*_ = 4.84*μm* (solid black line in Fig 1A), well within the SD region (Fig 1D). The crossover to the SD region is more prominent for higher *K* ^+^ in the bath solution (dashed black line in Fig 1A).

**Fig 2:**
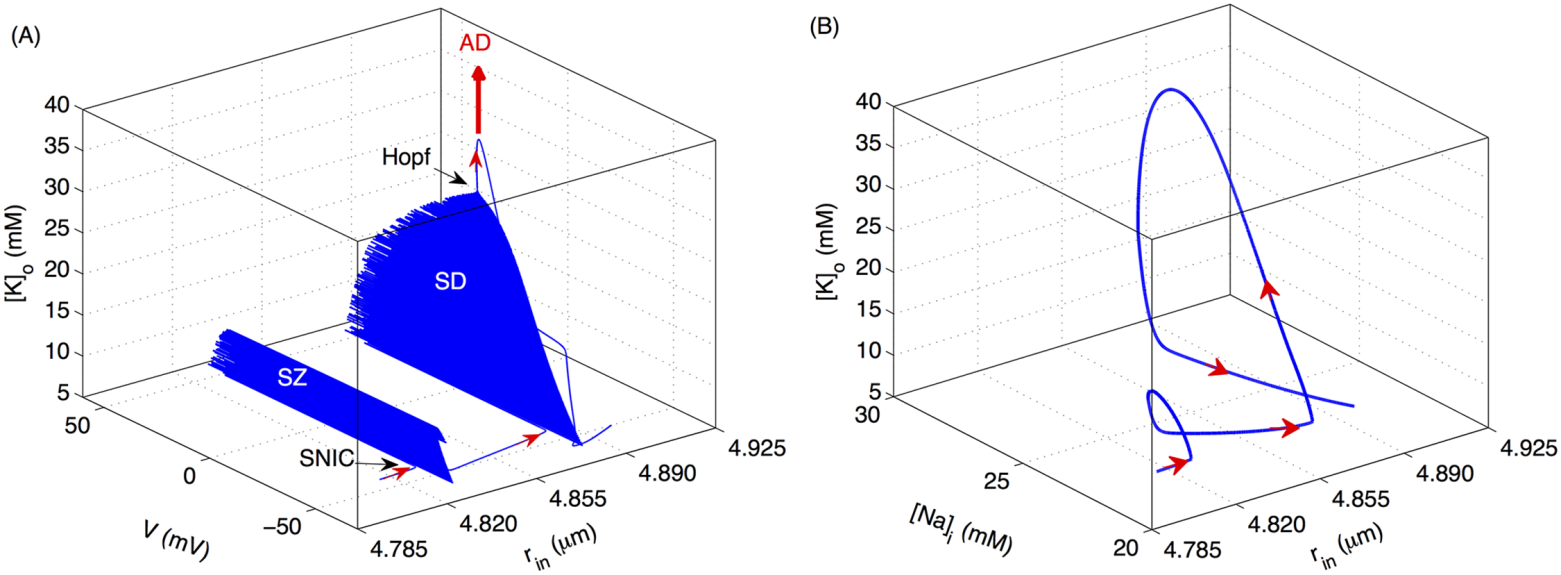
Transition of the cell from SZ to SD due to cell swelling. (A) Changes in membrane potential, [*K*]_*o*_, and *r*_*in*_ of the cell as it transitions from SZ to SD. (B) is from the same simulations as in (A) with the horizontal axis representing [*Na*]_*i*_ instead of *V*. The thick arrow indicates that for higher [*K*]_*o*_, whether due to higher *K* ^+^ exposure or hypoxia, the cell exhibits physiological AD as demonstrated below. Simulations based on the full model.

Fig 2 shows the pathway to the spontaneous transition from SZ to SD caused by cell swelling obtained by simulating the full model (Eqs 1–15). The arrows in Figs 2, 6, and 9 indicate the direction of the trajectory. Initially [*Na*]_*i*_ and [*K*]_*o*_ slowly build up leading to an increase in intracellular volume (hence a decrease in the relative extracellular space) and higher excitability of the cell. The microenvironment reaches a point where *V* makes a transition from steady state to limit cycle via a saddle node on invariant cycle (SNIC) bifurcation and the cell exhibits a SZ (Fig 2A). After exiting the SZ state, the cell does not have sufficient time to reverse the swelling (normally pumps would restore ionic gradients reversing the swelling) and the cell exhibits a SD event by entering a second periodic orbit via a SNIC, with progressively decreasing amplitude followed by entrance into a depolarized state via a Hopf bifurcation. Fig 2B shows the variations in [*Na*]_*i*_ during this transition. The thick arrow in Fig 2A indicates that the cell would go into a depolarized state similar to ischemia-induced AD if [*K*]_*o*_ increases further either due to swelling or lack of *O*_2_. This point will be further elaborated later in this paper.

**Fig 3:**
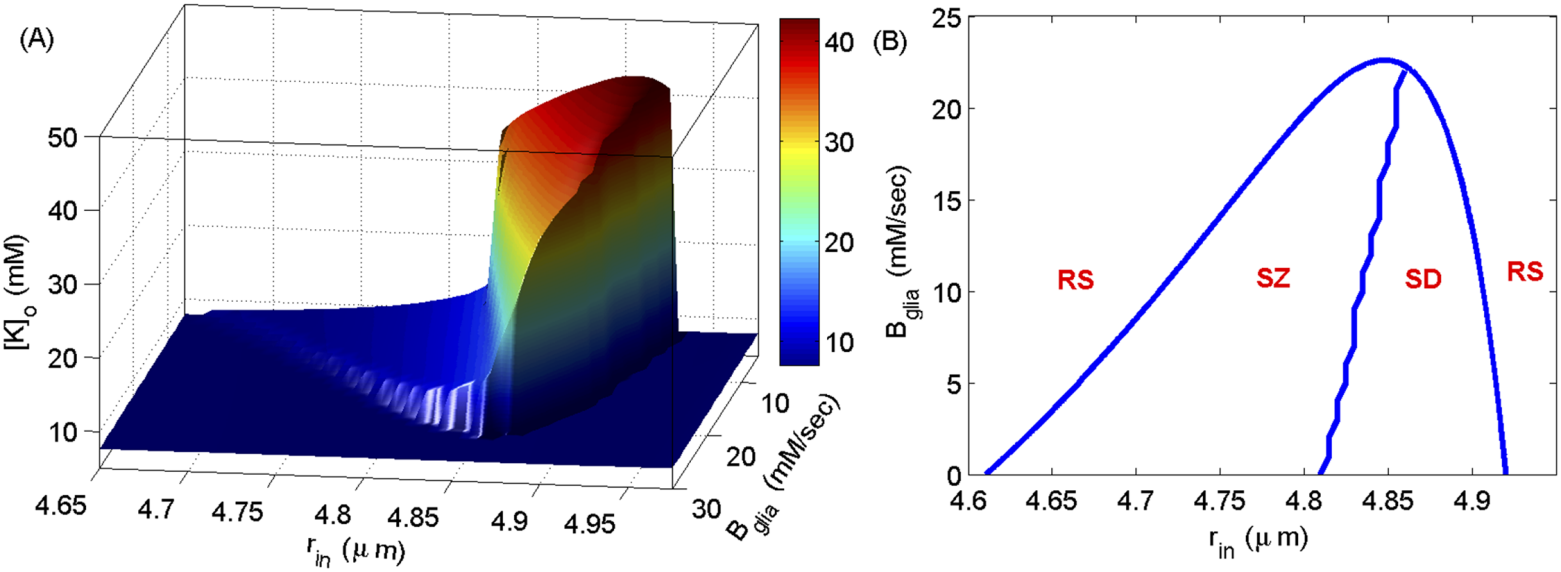
Two parameters bifurcation diagram for [*K*]_*o*_. (A) The maximum of [*K*]_*o*_ oscillations as a function of *r*_*in*_ and *B*_*glia*_. (B) The regions where the cell shows resting state (RS), SZ, and SD behaviors. We simulate Eqs (1, 3, 5, 7, 10) and take volume as a bifurcation parameter.

What physiological mechanisms help to regulate the brain so that most of the time, even in people with chronic recurring seizures and migraines, their brains are operating normally? To begin to address this question we show a two-parameters bifurcation diagram for [*K*]_*o*_ in Fig 3 where the two parameters are *r*_*in*_ and glial *K* ^+^ buffering strength, *B*_*glia*_. In Fig 3A, we show the maximum of [*K*]_*o*_ as a function of *r*_*in*_ and *B*_*glia*_. As is clear, for weaker glial buffering the cell’s behavior changes as a function of *r*_*in*_ in the same manner as in Fig 1. However, for stronger glial buffering (*B*_*glia*_ > 21mM/sec) the cell becomes biased towards SZ behavior and is less likely to go to SD. The stronger glial buffering siphons away [*K*]_*o*_ fast enough so that the cell recovers from swelling during SZ before entering SZ again. For even stronger glial buffering, the cell neither shows SZ nor SD behavior. This point is emphasized further in Fig 3B where we show the three regions described in Fig 1 as a function of *r*_*in*_ and *B*_*glia*_. For larger *B*_*glia*_, both SZ and SD regions disappear. This demonstrates that glial *K* ^+^ buffering can play a neuroprotective role against SZ and SD behaviors and, if strong enough, will constrain the neuron to physiological dynamics. From our previous work, we know that extracellular diffusion shares similar dynamical properties with the glial buffer (see, e.g., Fig 7 in [18]).

**Fig 4:**
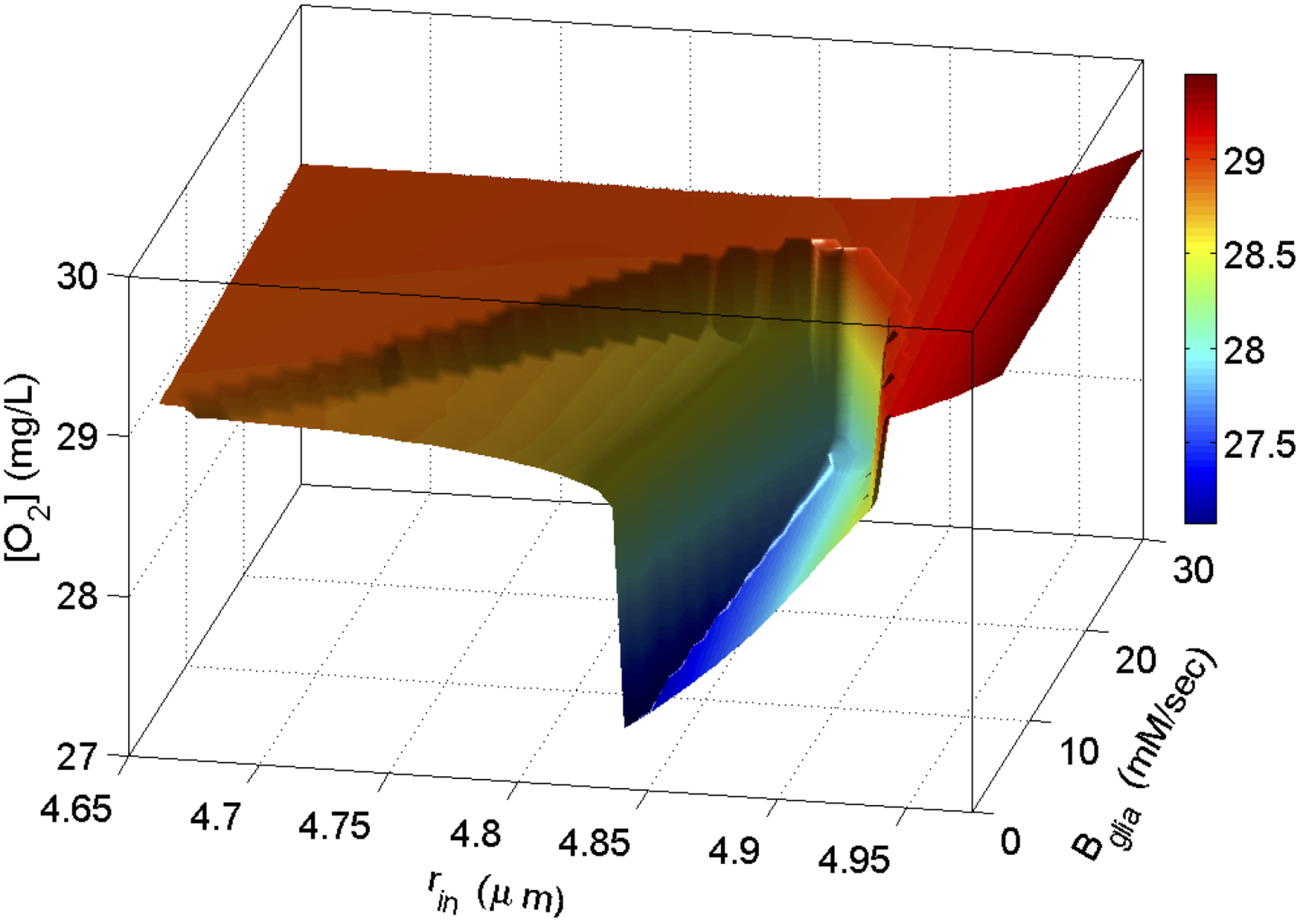
Two dimensional bifurcation diagram for local available *O*_2_ from simulations in Fig 3. The vertical axis shows the minimum of [*O*_2_] during oscillations.

A two parameter bifurcation diagram for [*O*_2_] from simulations in Fig 3 shows that the resting state at large *r*_*in*_ (right side in Fig 3B) is energetically favorable as compared to the one at small *r*_*in*_ (left side in Fig 3B) (Fig 4). Interestingly, the local *O*_2_ consumption during SD in a cell with smaller radius is much larger than the consumption in a cell that has swollen. This indicates that although the [*K*]_*o*_ changes during SD in smaller cells are smaller, *Na* ^+^−*K* ^+^ exchange pumps consume more energy to restore physiological [*K*]_*o*_ and [*Na*]_*i*_ in these cells due to their relatively larger extracellular reservoirs. A cell with an infinitesimally small extracellular space requires an infinitesimally small ionic flux to substantially change its extracellular potential, and an infinitesimally small work load on the membrane pumps to restore those gradients. As mentioned above, the bifurcation diagrams for [*Na*]_*i*_, [*K*]_*i*_, and [*Na*]_*o*_ (similar to Fig 1A) (not shown) show that their behavior contrasts with [*K*]_*o*_. That is, the amplitudes of [*Na*]_*i*_, [*K*]_*i*_, and [*Na*]_*o*_ oscillations decrease with increasing *r*_*in*_. Because the pumps are stimulated by [*Na*]_*i*_ and [*K*]_*o*_ (Eq 3), higher concentrations of [*Na*]_*i*_ in a smaller cell overwhelms the relatively smaller concentrations of [*K*]_*o*_ and the *Na* ^+^ − *K* ^+^ exchange pumps increase their rates accordingly to rearrange those ions (Fig 4). It is important to point out that the amount of oxygen available to the cell also acts as a bifurcation parameter causing the cell to transition between different states [18, 33].

### Bifurcation analysis of different neuronal behaviors

**Fig 5:**
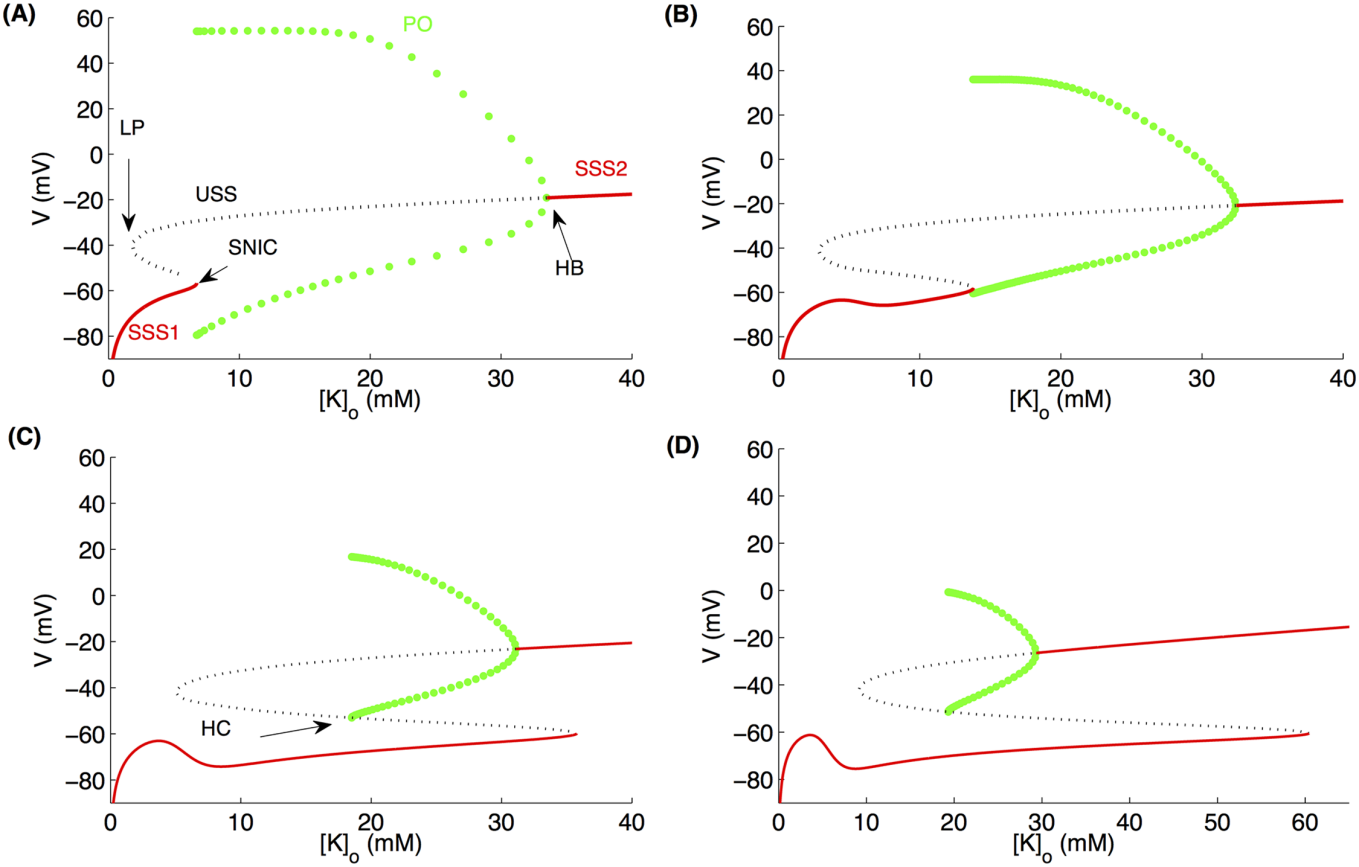
Bifurcation diagrams showing *V* as a function of [*K*]_*o*_ for four different [*Na*]_*i*_ values. (A) [*Na*]_*i*_ = 18*mM*, (B) [*Na*]_*i*_ = 24*mM*, (C) [*Na*]_*i*_ = 29*mM*, and (D) [*Na*]_*i*_ = 32*mM*. For the simulation in this figure, [*Cl*]_*i*_ = 8*mM* and normal [*O*]_2_ of 30 mg/L is used. SSS, USS, LP, PO, and HC stand for stable steady state, unstable steady state, limit point, periodic orbit, and homoclinic bifurcation respectively.

To gain further insights into the mechanisms behind the three behaviors in Fig 1C-1E exhibited by the cell, we performed a bifurcation analysis of the model fixing the slower variables [*K*]_*o*_, [*Na*]_*i*_, [*Cl*]_*i*_, [*O*_2_], and volume (*Vol*). In Fig 5, we show the maxima and minima of *V* as a function of [*K*]_*o*_. For small [*K*]_*o*_ values, the cell remains in stable steady state (SSS1; red line on the left) which collides with an unstable steady state (USS; black dotted line) giving rise to a periodic orbit (PO; green circles) through a SNIC bifurcation (Fig 5A). The unstable steady state gains stability (SSS2; red line on the right) through a Hopf bifurcation (HB) where the membrane potential of the cell is locked into a depolarized state. The SNIC moves to the right as we increase [*Na*]_*i*_ (Fig 5B) and bifurcates into a saddle homoclinic (HC) bifurcation (Fig 5C,5D).

**Fig 6:**
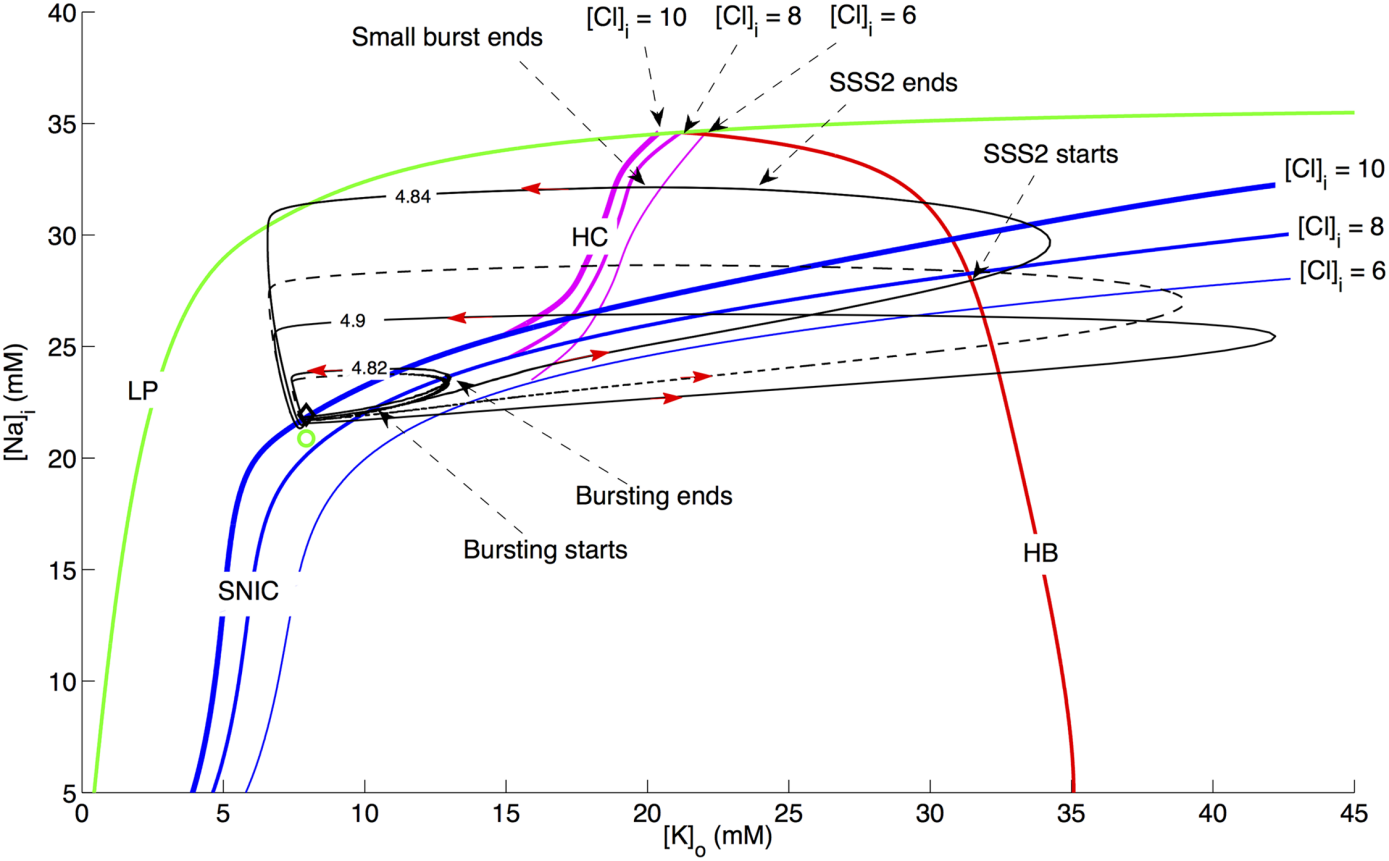
Two-parameter bifurcation diagram. The locations of HB (red), SNIC (blue), HC (pink), and LP (green) with [*K*]_*o*_ and [*Na*]_*i*_ as bifurcation parameters for three different [*Cl*]_*i*_ values and normal [*O*]_2_ of 30 mg/L. The locations of SNIC and HC change with intracellular *Cl* ^−^ as shown for [*Cl*]_*i*_ = 6*mM*, 8*mM*, and 10*mM* where the thickness of the blue and pink lines represents increasing [*Cl*]_*i*_ value. The position of HB and LP does not change significantly and is therefore only shown for [*Cl*]_*i*_ = 8*mM*. The three solid black traces represent the limit cycles from the full model (Eqs 1, 3, 5, 7, 10), and fixed volume) for three *r*_*in*_ values shown in the figure in *μm* and correspond to the three behaviors in Fig 1C (with slightly larger r_in_), Fig 1D and 1E respectively. The red arrows show the direction of trajectories. The diamond and small circle represent steady states at *r*_*in*_ = 4.6*μm* and 4.95*μm* respectively. The black dashed curve is a 2D version of Fig 2B and is shown for comparison.

The locations of special points, HB, SNIC, the limit point (LP) (Fig 5A), and HC (Fig 5C) as [*K*]_*o*_ and [*Na*]_*i*_ vary simultaneously are shown through a two-parameter bifurcation diagram in Fig 6. The limit cycles and steady states from the full (but with fixed volume) model at different *r*_*in*_ values are also shown in Fig 6. For clarity, we will restrict our discussion to the case of [*Cl*]_*i*_ = 8mM but the argument applies to the other values of [*Cl*]_*i*_ as well. During the SZ event like the one shown in Fig 1C (*r*_*in*_ = 4.82*μm*), the cell enters the periodic orbit from steady state on the left (SSS1 in Fig 5A) through a SNIC bifurcation and spikes for a few seconds before going back to SSS1. The [*K*]_*o*_ values when the trajectory crosses the SNIC bifurcation curve indicate the beginning and end of the burst in the time trace. Since the burst terminates at a SNIC, the frequency of the burst is low at the beginning and end of the burst. This limit cycle with bursts never generates [*K*]_*o*_ substantially above a physiological ceiling, about 12 mM experimentally [42] and about 13–14 mM in Fig 6, below which spikes and seizures are observed but not SD. Indeed, the model generates rather pure seizure dynamics with [*K*]_*o*_ below the ceiling.

At *r*_*in*_ = 4.84*μm* (Fig 1D), there is a substantial change in [*Na*]_*i*_ and [*K*]_*o*_ and the one-parameter bifurcation diagram goes through the transitions shown in Fig 5A-5D. The beginning of the first burst starts when trajectory crosses the SNIC curve in Fig 6 and terminates when it crosses the HB curve. Again, the frequency within the burst is low at the beginning. The amplitude shrinks to zero at the end as the trajectory passes through the HB curve. After the first burst terminates, the trajectory follows the stable branch of SSS2 (Fig 5) for a while before it crosses the HB curve again. The solution does not start to burst at this moment, because of the delayed loss of stability phenomenon (delayed HB) [43] that occurs when a parameter passes slowly through a HB point and the system’s response changes from a slowly varying steady state to a slowly varying oscillation. So, the trajectory traces the unstable branch of SSS2 for a while before it starts to burst between HB and HC curves. Also referred to as “delayed” or “memory effect”, this transition happens when the parameter is considerably beyond the value predicted from a straightforward bifurcation analysis which neglects the dynamic aspect of the parameter variation. This memory effect has been studied for different problems including the FitzHugh-Nagumo model [44]. The second burst terminates when the trajectory crosses the HC curve (notice the slight delay in the termination of the second burst for higher [*Cl*]_*i*_ values). At this moment, the solution drops to the lower stable branch SSS1. Since [*Na*]_*i*_ is approximately 30mM for the second burst, the amplitude of the second burst is smaller according to Fig 5. The delay also explains why the burst onset has a nonzero amplitude since the solution is away from the HB curve. The amount of delay depends on the time spent near the attracting branch of SSS2 (the loop from HB to HB).

The explanation for *r*_*in*_ = 4.84*μm* also applies to the case with *r*_*in*_ = 4.9*μm* (Fig 1E), except that the HB to HB trajectory loop is more dramatic than *r*_*in*_ = 4.84*μm*. The trajectory spends more time on the attracting branch of SSS2 that increases the delay. Here, the solution moves along the unstable branch through the entire HB-HC regime without oscillating. It finally loses stability, but since there is no stable limit cycle anymore, it can only drop to the lower stable branch of SSS1. SSS2 (the depolarized state of *V*) ends to the left of HC curve. The drop to SSS1 (end of depolarized state) is faster (as in Fig 1D) for a slightly smaller cell (for example when *r*_*in*_ = 4.88*μm*) because the trajectory is close to the SNIC-HC bifurcation point and hence the upper and lower branches are closer (not shown).

### Anoxic depolarization

**Fig 7:**
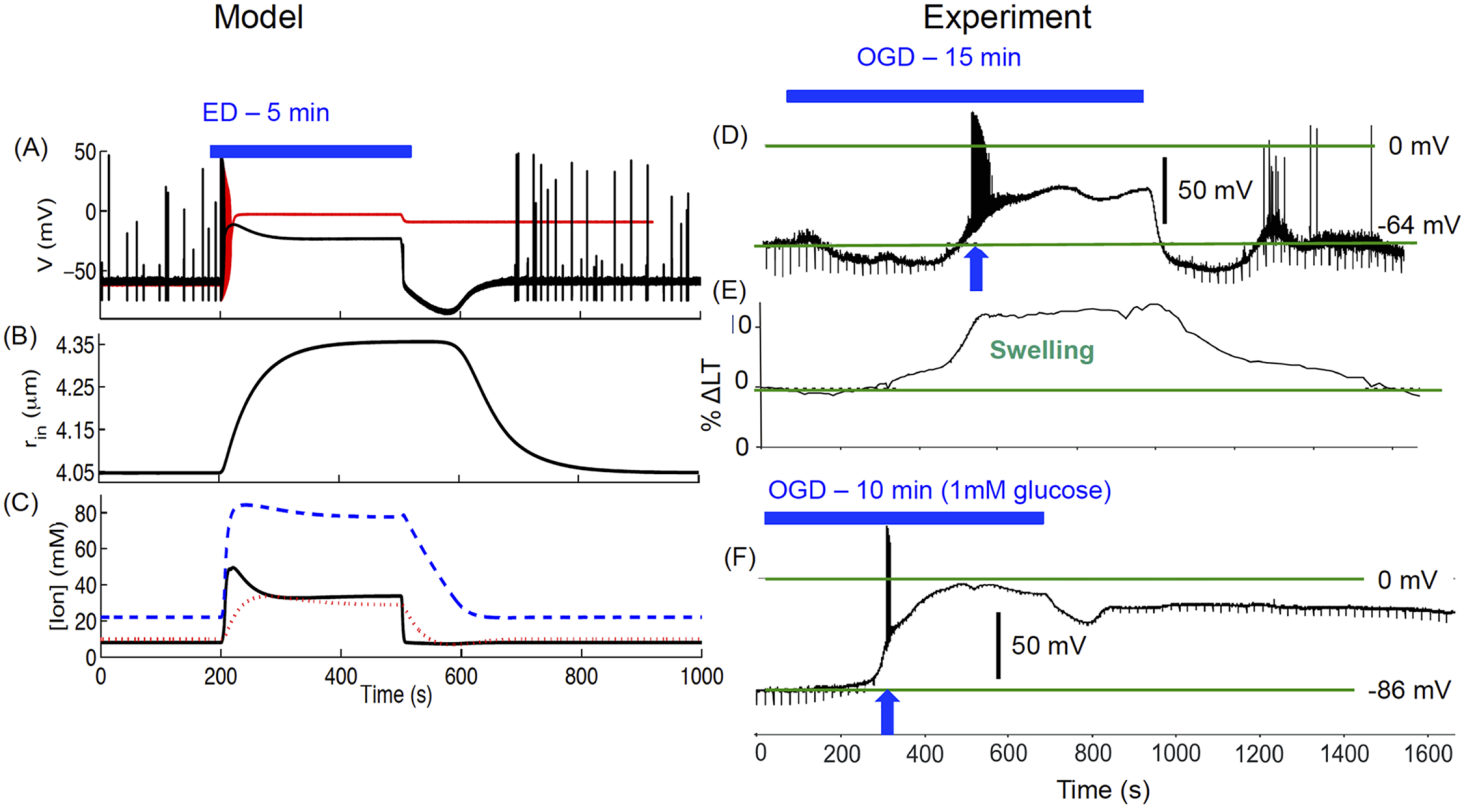
Model cell response to energy deprivation (ED, the model-equivalent of OGD). The cell exhibits AD in response to 5 min ED and returns back to normal behavior after ED ends (A). Change in cell radius (B) and ion concentrations (C) during ED. Solid, dashed, and dotted lines in (C) represent [*K*]_*o*_, [*Na*]_*i*_, and [*Cl*]_*i*_ respectively. The black line in (A) and all lines in (B, C) correspond to initial *r*_*in*_ = 4*μm*, while the red line in (A) is for initial *r*_*in*_ = 3*μm*. (D) shows experimental membrane potential of a magnocellular neuroendocrine cell (MNC) under 15 min OGD. Panel (E) shows the percent change in light transmittance (Δ*LT*) representing cell swelling, while (F) shows AD exhibited by a pyramidal neuron in neucortical layer V. (D-F) Data provided by David Andrew. (D-F) modified from [20] with permission American Physiological Society. Simulations based on the full model.

In addition to SZ, SD, and SZ-SD transition, our model closely reproduces AD. AD, the initial electrophysiological event during ischemia, is a front of depolarization that drains residual stored energy in compromised gray matter. Recently, Brisson and Andrew studied AD at the single cell level in neocortex and hypothalamus slices deprived of *O*_2_ and glucose [20]. They used two-photon microscopy to image cell swelling simultaneously with patch-clamp membrane potential measurements in the OGD condition. Although, our model does not have the glucose component, it behaves in the same manner as that observed experimentally during energy substrate deprivation. We induced energy deprivation (ED) in the model by putting oxygen in the perfusion solution, [*O*_2_]_∞_, equal to zero, which is model-equivalent to OGD or ischemia-induced AD [45]. In Fig 7A we show the cell behavior in response to 5 min ED. During AD, the cell swells qualitatively in the same manner as observed in experiments (Fig 7B and 7E). The extra- and intracellular ion concentrations go through a massive redistribution during AD (Fig 7C). Consistent with the experimental observations (shown in panels D-F), the cell depolarizes and swells significantly during AD. The behavior of the model-cell is reminiscent of the magnocellular neuroendocrine cell (MNC) in the hypothalamus with weak AD that depolarizes only transiently close to 0*mV* (Fig 7D). However, as we change the initial radius of the cell from 4.0*μm* to 3.0*μm*, it exhibits strong AD, approaching a steady Donnan equilibrium of −2mV (red line in Fig 7A). This behavior is reminiscent of strong AD in pyramidal neurons from neucortical layer V where the cell’s membrane depolarizes to near 0mV during OGD and does not recover to the pre-OGD state when *O*_2_ and glucose supply is restored (Fig 7F). Our result sheds light on the importance of cell size and packing density in the strength of AD and the potential damage to the cell where the membrane potential is permanently brought into a depolarized state with no recovery to the pre-ED state.

During the modeling of AD, we made some additional observations. The main conclusion about the transition between SZ and SD states qualitatively remain the same for fixed *Cl* ^−^ concentrations. However, the cell cannot sustain a prolonged depolarization similar to AD for fixed *Cl* ^−^ concentrations (S3(A) Fig), confirming an important role of *Cl* ^−^ in AD [46]. A similar behavior is observed when we maintain the normal *K* ^+^ diffusion between extracellular space and bath solution (S3(B) Fig). Because diffusion of ions within the extracellular space is a function of the size and geometry of extracellular space [47], we needed to make diffusion a function of *O*_2_ [33] so that there is decreased exchange of *K* ^+^ between bath or blood vessels and extracellular space when the cell is exposed to reduced oxygen. In addition, we had to make the *K* ^+^ glial buffering a function of *O*_2_ to reproduce AD (as in [48]). Several observations support these assumptions. A significant portion of [*K*]_*o*_ is buffered by astrocytes through ATP-dependent *Na* ^+^ − *K* ^+^ pumps that do not function in the absence *O*_2_ and glucose. In the absence of *O*_2_, astrocytes attempt to buffer the increased extracellular *K* ^+^ by switching to anaerobic glycolysis and swell substantially [49], further restricting *K* ^+^ diffusion and limiting glial energy reserves. Astrocytic inward rectifying *K* ^+^ channels (*K*_*ir*_) also contribute to *K* ^+^ siphoning, gating through interaction with G-protein coupled receptors pathway that is dependent on ATP (see [50] for a review on *K*_*ir*_ channels). Similarly, *Na* ^+^ − *K* ^+^ − *Cl* ^−^ cotransporters (NKCC) that are found in astrocytes play a significant role in transferring *K* ^+^ (together with *Na* ^+^ and *Cl* ^−^) from extracellular space to astrocytes and are dependent on ion gradients [51] and thus indirectly on ATP. Hence ATP plays a crucial role in these pathways that would be disrupted in the absence of *O*_2_, leading to reduced *K* ^+^ buffering.

**Fig 8:**
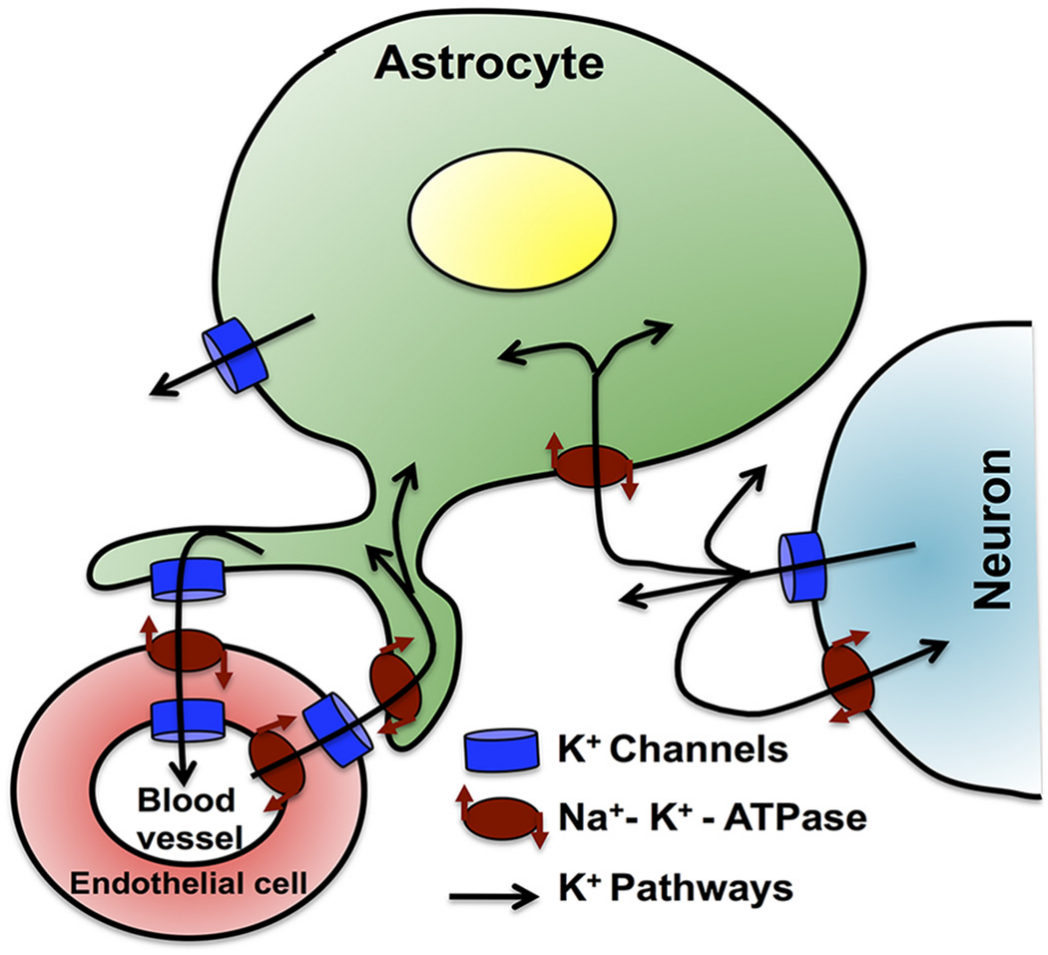
*K* ^+^ exchange at the blood-brain barrier. Extracellular *K* ^+^ absorbed by astrocytes is transferred to the blood vessels through a sequential functioning of *K* ^+^ channels and *Na* ^+^ − *K* ^+^ exchange pumps at the junction between astrocytes and endothelial cells surrounding the blood vessel lumen. A reverse process transfers *K* ^+^ from blood vessels to astrocytes. Omitted from this picture is the *K* ^+^ exchange between astrocytes through gap junctions.

The other important effect is the reduced transport of ions at glial end-feet adjoining the pericapillary space. The two-way *K* ^+^ trafficking at the blood-brain barrier occurs at the junctions between astrocytic end-feet and blood vessels (see for example [52]). Astrocytes release *K* ^+^ next to tight junction sealed endothelial cells in blood vessels. *Na* ^+^ − *K* ^+^ pumps transfer that *K* ^+^ to the endothelial cells and it is then delivered into the blood vessels through *K* ^+^ channels. A reverse process transfers *K* ^+^ from blood vessels to astrocytes and finally to neurons (Fig 8). The lack of ATP in AD would disrupt this pathway, consequently impairing the *K* ^+^ diffusion between blood vessels and extracellular space.

**Fig 9:**
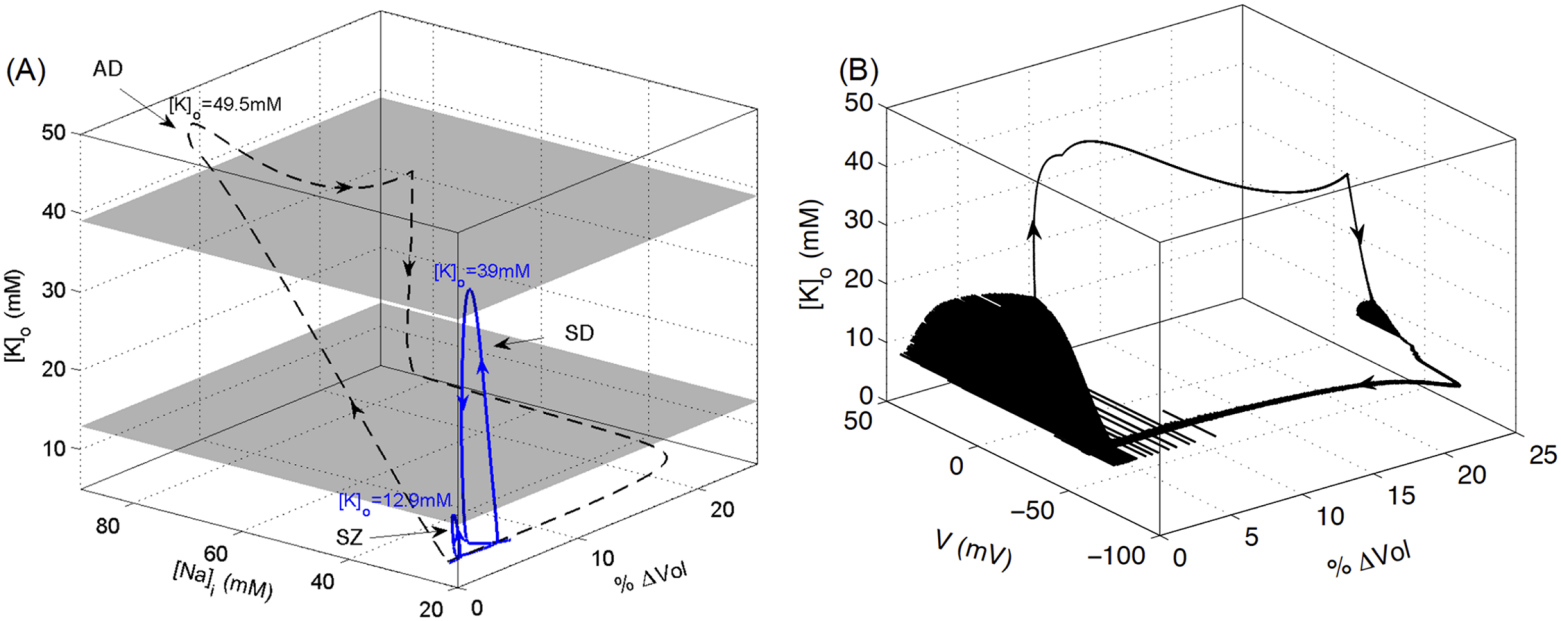
A comparison of microenvironmental changes during SZ, SD, and AD. (A) Solid and dashed lines represent the simulations in Figs (2) and (7) respectively. %Δ*Vol* represents the percent change in volume and is defined as $((r_{in,ins}^{3}-r_{in,ss}^{3})/r_{in,ss}^{3})\times 100$, where *r*_*in*,*ss*_ and *r*_*in*,*ins*_ represent initial steady state (*ss*) and instantaneous (*ins*) radius of the cell respectively. The peak [*K*]_*o*_ values during SZ, SD, and AD are shown for comparison. The lower grey plane corresponds to the physiological ceiling for [*K*]_*o*_ [42], here calculated at 12.9 mM in the model, whereas the upper plane, which separates SD from AD, is found at 39 mM [*K*]_*o*_. (B) is from the same simulations as shown by the dashed line in (A) and shows the change in membrane potential along with [*K*]_*o*_ and volume. Simulations based on the full model.

In Fig 9, we show the pathways of microenvironment changes leading to AD. Immediately after setting *O*_2_ to zero, there is a rapid increase in [*K*]_*o*_ and [*Na*]_*i*_ followed by a slow increase in intracellular volume (Fig 9A). [*K*]_*o*_ drops slightly from its peak value once it enters the AD due to the slight delay in the [*Cl*]_*i*_ rise (see Fig 7C). After the initial drop, [*K*]_*o*_ stabilizes at fixed value when [*Cl*]_*i*_ plateaus at its peak value. Once *O*_2_ is restored, [*K*]_*o*_ is restored to normal values followed by slow restoration of [*Na*]_*i*_ and intracellular volume. The blue curve is replotted from Fig (2B) to compare the cell dynamics during SZ, SD, and AD. The change in the membrane potential along with [*K*]_*o*_ and percentage change in the cell volume during the simulation represented by the dashed line in Fig 9A is shown in Fig 9B. The gray planes in Fig 9A indicate the approximate regions for SZ, SD, and AD. The cell exhibits SZ below the physiological [*K*]_*o*_ ceiling represented by the bottom plane, SD between bottom and top planes, and AD above top plane. The position of these planes will change with the size and density of neurons.

## Discussion

This is, to our knowledge, the first detailed study of the effects of cell volume on neuronal dynamics. We employed a recently discovered unification framework, where extending the Hodgkin-Huxley equations for mammalian neurons with energy balance and conservative principles demonstrated that a broad variety of neuronal states lie along a continuum of the repertoire of the neuronal membrane [33]. Using a variety of model simplifications, we were able to perform detailed bifurcation analyses that explained the full model effects as a function of volume itself as an order parameter. We can now better understand and unify a range of effects and factors that are critical in the transitions to the pathological states of SZ, SD, and AD.

Our study of the role of volume as an order parameter revealed a dynamical definition for the experimentally described physiological ceiling that separates seizure from SD activity [42]. Furthermore, we have delineated and predict a second ceiling, one that demarcates SD from AD. Our observations unveil a new way of investigating neuronal behavior where different states need not be treated separately but rather as a dynamical continuum of the neuronal membrane potential, ion concentrations, metabolic energy, and volume.

Previous experiments support our observations about the volume as an order parameter regulating the neuronal excitability. Exposure of brain cells to hyposmolality and the resultant shrinkage of extracellular space at clinically relevant levels promotes epilepiform activity in hippocampus and neocortex [53–55] and clinically [5]. Hyperosmolality (dehydration), on the other hand inhibits epilepiform activity. Mannitol, which reverses the hyposmolar state, abolishes synchronous neuronal bursting in the dentate gyrus and CA1 area of the hippocampus [4]. Furthermore, hyposmolality increases the amplitude of evoked field potentials and excitatory postsynaptic potentials recorded intracellularly in rat neocortical slices [7]. Conversely, mannitol-induced hyperosmolality reverses these features [7]. Similarly, decreasing the extracellular osmotic pressure converted non-bursting neurons to bursting neurons and decreased the stimulus requirements for evoking burst firing in native bursters, while increasing extracellular osmotic pressure suppressed burst firing [56].

Glial reactivity and scarring is a prominent feature of a broad variety of brain pathologies [57], and is prominent in epilepsy [58]. We found that when glial *K* ^+^ buffering is impaired, the cells can swell enough to cause transition from SZ to SD. Previous observations by Foley et al. [59] support this mechanism. The excitability of neurohypophysial neurons due to accumulating *K* ^+^ in the extracellular space decreased significantly by increasing the size of interstitial space [59]. The decrease in action potential amplitude (showing the cell’s transition towards a depolarized state (SSS2)) in control cells in response to a train of 40 stimuli at 25Hz, diminished when a hypertonic solution containing 100mM sucrose was added to the normal ringer solution perfusate [59]. The membrane-impermeable sucrose increases extracellular osmotic pressure causing neurons to shrink. A similar protocol could be used to test the predictions of our model. Our hypothesis is that adding hypertonic solution to the tissue going though SD would transition the cell to SZ and possibly to the resting state. Taking glial swelling into account would make the shrinkage of interstitial space more dramatic, further supporting our claim that the reduction in extracellular space can be strong enough to cause the cell to transition from SZ to SD. Our results also highlight the importance of glial *K* ^+^ buffering strength in pathological states and shows that the cell is less likely to go into SZ and SD and the transition between the two when glia are functioning efficiently.

The different time-scales in our model provide a parsimonious explanation for the transition between SZ and SD states in Fig 1A. As mentioned above, there are four different time-scales in our model: fast (variables *V*, *n*, and *h*; Eq (1)), intermediate ([*K*]_*o*_, [*Na*]_*i*_; Eqs (3, 7), slow ([*O*_2_], [*Cl*]_*i*_; Eqs (5, 10)), and infra-slow (*Vol*; Eq (14)). Strictly speaking, the ultra-fast m-gate (*m*) if modeled with the rate equation instead of the steady state approximation can be considered as a fifth time-scale. We believe that this could underlie the dramatic increase in the amplitude of [*K*]_*o*_ oscillations—reminiscent of canard explosion—which is a feature of a slow-fast system such as ours [60]. Testing this claim using geometric singular perturbation theory is beyond the scope of this study and will be the subject of future research.

In addition to the spontaneous transition between SZ and SD, our model closely reproduces AD, the hyperpolarization after *O*_2_ is restored, and the cell swelling in AD. The close resemblance of our results to the experimental data makes our model a good candidate for future studies to understand hypoxia and ischemia and ways to protect against metabolic insults.

As pointed out above, Brisson et al. [20, 21] emphasized the importance of variable pump rates in the stronger AD observed in the upper brain regions as compared to the lower brain regions in response to OGD. Our study shows that the cell volume and microenvironment play an important role in the strength of AD (Fig 7A). Our preliminary phase space analysis for the cell’s failure in the higher brain regions to recover from AD reveals that the cell volume affects [*K*]_*o*_ and [*Na*]_*i*_ in such a way that they lead to lower pump activity and consequently the cell’s failure to recover from AD after *O*_2_ and glucose is restored. A complete phase space analysis of the recovery failure from AD is beyond the scope of this paper and the subject of our future research.

This current model is a simplified picture of a very complex reality. Although *Na* ^+^ − *K* ^+^-ATPase consumes 91% of total available *O*_2_ [61], other pathways such as *Ca* ^2+^-ATPase and synaptic communication expend significant amounts of metabolic energy which is not taken into account in the current model. Similarly, the clearance of excessive *K* ^+^ accompanied by *Cl* ^−^ uptake and *Na* ^+^ expulsion by astrocytes in face of high neuronal activity would lead to significant dilation of astrocytes [26–28] during the pathological states discussed in this paper, which is missing from our model. Also missing from the model is the dynamic intra- and extracellular *Ca* ^2+^ concentrations, which are suspected to play a crucial role in neuronal excitotoxicity (see for example, chapter 4 of [1]). Osmotic pressure also affects the re-depolarizing component of spike after-depolarization and apparent membrane time-constant that are attributed to changes in the persistent Na^+^ current [56]. These factors are important for developing a more comprehensive understanding of the pathological states discussed in this paper, and is the subject of future research.

To conclude, our finding further explored a unified mechanism that accounts for SZ, SD, and AD. The detailed biophysical models that take neuronal microenvironment such as ion concentrations, glia, blood vessels, metabolic energy requirements, and volume homeostasis into account will provide a better understanding of these conditions and may lead to unified therapies for SZ, SD, and possibly ischemic stroke-like injury prevention.

## Methods

Our model builds on our previous work [15, 18, 33, 62]. We consider a spherical cell of radius *r*_*in*_ placed inside a spherical shell of fixed radius *r*_*tot*_ = 5*μm*. So by changing *r*_*in*_, we are in effect changing the ratio of intra- to extracellular volume. The schematic of the model is shown in Fig 1 of [33].

### Hodgkin-Huxley type equations

The membrane potential *V* of the cell is modeled with the following set of modified Hodgkin-Huxley type equations [15, 63]

$$\begin{array}{r} C\frac{dV}{dt}=I_{Na}+I_{K}+I_{L}-I_{pump}+I_{rand},\\ I_{Na}=-g_{Na}m^{3}h\left(V-V_{Na}\right),\\ I_{K}=-g_{K}n^{4}\left(V-V_{K}\right),\\ I_{L}=I_{KL}+I_{NaL}+I_{ClL}=-g_{KL}\left(V-V_{K}\right)-g_{NaL}\left(V-V_{Na}\right)-g_{ClL}\left(V-V_{Cl}\right),\\ dq/dt=\alpha_{q}\left(1-q\right)-\beta_{q}q,\;q=m,n,h. \end{array}$$ (1)

Where *n* ^4^ and *m* ^3^ *h* represent the gating variables for delayed rectifier potassium (*I*_*K*_) and transient sodium (*I*_*Na*_) currents. The leak current (*I*_*L*_) has three components: *K* ^+^ (*I*_*KL*_), *Na* ^+^ (*I*_*NaL*_), and chloride (*I*_*ClL*_) leak. *I*_*pump*_ is the net current due to the ATP-dependent pump that extrudes 3 *Na* ^+^ for bringing 2 *K* ^+^ in. A random current (*I*_*rand*_) representing the background input from the other neurons is included in some simulations and is modeled by a zero mean Gaussian processes. The meaning and values of parameters used in the model are given in Table 1.

**Table 1:** Model Parameters.

Parameter	Value	Description
*C*	1*μ* F/cm^2^	Membrane capacitance
${\bar{g}}_{Na}$	100mS/cm^2^	Conductance of Sodium Current
${\bar{g}}_{K}$	40mS/cm^2^	Conductance of potassium current
${\bar{g}}_{KL}$	0.05mS/cm^2^	Conductance of potassium leak current
${\bar{g}}_{NaL}$	0.02mS/cm^2^	Conductance of sodium leak current
${\bar{g}}_{ClL}$	0.05mS/cm^2^	Conductance of chloride leak current
*ϕ*	3sec^−1^	Time constant of gating variables
*β*	varies	Ratio of intra- to extracellular volume of the cell
*ρ*	3.85*μA*/*cm* ^2^	Maximum pump strength
*B*_*glia*_	5mM/sec, varies in Figs 3, 4	Maximum strength of glial uptake
*ϵ*_*O*_	0.34*sec* ^−1^	Diffusion constant of *O*_2_
*O*_2,∞_	30 *mg*/*L*	*O*_2_ concentration in the perfusion solution
*α*	6	conversion factor from mM/sec to mg/L/s
*ρ*_*KCC*_	0.5 mM/sec	maximum strength of *K* ^+^ − *Cl* ^−^ cotransporter

The rate equations for the gating variables are [64]

$$\begin{array}{ccc} \alpha_{m} & = & \frac{0.1(V+30)}{1-exp(-0.1(V+30))},\\ \beta_{m} & = & 4exp(-\frac{V+55}{18}),\\ \alpha_{n} & = & \frac{0.01(V+34)}{1-exp(-0.1(V+34))},\\ \beta_{n} & = & 0.125exp(-\frac{V+44}{80}),\\ \alpha_{h} & = & 0.07exp(-\frac{V+44}{20}),\\ \beta_{h} & = & \frac{1}{1+exp(-0.1(V+14))}. \end{array}$$ (2)

We will use the instantaneous steady-state form of *m*, i.e. *m* = *α*_*m*_/(*α*_*m*_ + *β*_*m*_) [65].

### Ion concentration dynamics

The ion current equations are augmented with dynamic intra- and extracellular concentrations of *K* ^+^, *Na* ^+^, and *Cl* ^−^. These concentrations are modulated by the neuron’s intrinsic ionic currents, *Na* ^+^ − *K* ^+^ pump current, and *K* ^+^ − *Cl* ^−^ cotransporters. The glial buffering and diffusion into the microenvironment of the cell (from the bath solution in slice preparation and vasculature *in vivo*) also modulate the *K* ^+^ concentration in the extracellular space. The ion concentrations inside and outside the cell are coupled to the membrane voltage equations via the Nernst equation. The rate equations for *K* ^+^ and *Na* ^+^, *O*_2_, and *Cl* ^−^ concentrations and the rational for different terms within these equations are described in detail in [15, 66–68], [18], and [33] respectively and are summarized below.

Given *I*_*K*_, *I*_*KL*_, *I*_*pump*_, diffusion of *K* ^+^ to the microenvironment (*I*_*diff*_), glial buffering (*I*_*glia*_), and *K* ^+^ − *Cl* ^−^ cotransporters (*I*_*KCC*_), the extracellular *K* ^+^ dynamics, [*K*]_*o*_, can be represented in the model as

$$\begin{array}{ccc} \frac{d{\left[K\right]}_{o}}{dt} & = & \frac{1}{\tau }(\gamma \beta \left(I_{K}+I_{KL}-2I_{pump}\right)-I_{diff}-I_{glia}+I_{KCC}),\\ I_{pump} & = & 𝓕\left(\left[O_{2}\right]\right)\rho (\frac{1}{1+exp(\left(25-{\left[Na\right]}_{i}\right)/3)})(\frac{1}{1+exp(8-{\left[K\right]}_{o})}),\\ I_{diff} & = & 𝓕\left(\left[O_{2}\right]\right)\epsilon_{K}\left({\left[K\right]}_{o}-{\left[K\right]}_{o,\infty }\right),\\ I_{glia} & = & 𝓕\left(\left[O_{2}\right]\right)\frac{B_{glia}}{1+exp(\left(18-{\left[K\right]}_{o}\right)/2.5)}, \end{array}$$ (3)

where *τ* = 1000 is used to convert seconds to milliseconds. *γ* converts ionic current to concentration rate of change and is calculated using $\gamma =\frac{A}{F\times Vol}$ [15], where *A*, *Vol*, and *F* represent cell area, volume, and Faraday constant respectively. *β* is the intra- to extracellular volume ratio. *γ* and *β* change when the volume and surface area of the cell change. In simulations where volume is treated as a dynamical variable, both *γ* and *β* change dynamically. Modeling glial *K* ^+^ buffering by a rate equation as done in [16] qualitatively did not change our results. Similarly, making *I*_*diff*_ a function of *β* explicitly as done previously [33] did not change our conclusions (but see the discussion in “Anoxic depolarization” section).

The *Na* ^+^ − *K* ^+^ pump is modeled as a product of sigmoidal functions, *ρ* is the maximum pump strength, and [*Na*]_*i*_ is the intracellular *Na* ^+^ concentration [15]. Each sigmoidal term saturates for high values of [*Na*]_*i*_ and [*K*]_*o*_ respectively. The ATP required to keep the pump running depends on the local *O*_2_ availability. The ATP concentration and hence the pump strength decreases as the cell depletes its local *O*_2_ reservoir. We use a sigmoid function 𝓕([*O*_2_]) to model the *O*_2_ concentration ([*O*_2_])-dependence of the pump activity [18]

$$\begin{array}{ccc} 𝓕(\left[O_{2}\right]) & = & \frac{1}{1+exp(\left(16-\left[O_{2}\right]\right)/4)}. \end{array}$$ (4)

The justification for the [*O*_2_]-dependence of *I*_*diff*_ and *I*_*glia*_ terms is elaborated in [33] and further discussed in the Results section. For simplicity, we use the same functional form as in *I*_*pump*_ for the [*O*_2_]-dependence of *I*_*diff*_ and *I*_*glia*_. Using separate functions for the [*O*_2_]-dependence of these three fluxes as in [33] does not qualitatively change our results.

The local *O*_2_ concentration is controlled by the activity of the *Na* ^+^ − *K* ^+^ pump and diffusion of *O*_2_ from the bath solution (or vasculature) to the extracellular space. Thus the rate equation for [*O*_2_] as developed in [18] based on [*O*_2_] imaging experiments [17] is given as

$$\begin{array}{c} \frac{d[O_{2}]}{dt}=\frac{1}{\tau }(-\alpha \gamma I_{pump}+\epsilon_{O}\left({\left[O_{2}\right]}_{\infty }-O_{2}\right)). \end{array}$$ (5)

Where [*O*_2_]_∞_ is the oxygen concentration in the perfusion solution with a normal range of 30 − 32*mg*/*L* when aerated with 95%*O*_2_ and 5%*CO*_2_ at 32–34°*C*. *α* is a conversion factor that converts pump current in mM/s to *O*_2_ concentration change in mg/L/s and the diffusion constant of *O*_2_ (*ϵ*_*O*_) is obtained from Fick’s law [18].

[*K*]_*o*,∞_ in the diffusion equation (Eq 3) is the *K* ^+^ concentration in the nearby reservoir. Physiologically, this would correspond to either the bath solution in a slice preparation, or the vasculature in the intact brain (noting that [*K*]_*o*_ is kept below the plasma level by trans-endothelial transport). In physiological conditions, [*K*]_*o*,∞_ is set equal to 4mM. We use a previously published value for the diffusion constant of [*K*]_*o*_ to the nearby reservoir *ϵ*_*K*_ [66] which was obtained from Fick’s law. That is, *ϵ*_*K*_ = 2*D*/Δ*x* ^2^, where *D* = 250 × 10^−6^cm^2^/sec is the *K* ^+^ diffusion constant in neocortex [69], and Δ*x* ≈ 20*μ*m for brain reflects the average distance between neurons and capillaries [70].

Both active and passive *K* ^+^ uptake into glia is incorporated into a simplified single sigmoidal response function that depends on extracellular *K* ^+^ concentration with *B*_*glia*_ representing the buffering strength [15]. Two factors allow the glia to provide a nearly insatiable buffer for the extracellular space. The first is the large size of the glial network. Second, the glial endfeet surround the pericapillary space, which through interaction with arteriole walls affecting blood flow, and transport of ions to the vascular space, amplifies the effective buffering capability of the glia [71–73].

Chloride is the primary permeant anion and its homeostasis is important for a range of neurophysiological processes. Neurons regulate intracellular chloride ([Cl^−^]_*i*_) through cation-chloride cotransporters, especially the *Na* ^+^/*K* ^+^/2*Cl* ^−^ cotransporter (NKCC1) and *K* ^+^/*Cl* ^−^ cotransporter (KCC2) [74]. In the embryonic and early postnatal brain, neurons show robust expression of NKCC1 but minimal expression of KCC2 [74]. In the mature brain, the expression of KCC2 increases, accompanied by a concurrent down regulation of NKCC1 expression [74]. KCC2 is important in maintaining low [Cl^−^]_*i*_, resulting in hyperpolarizing GABA responses. Since KCC2 operates close to its thermodynamic equilibrium: [*Cl* ^−^]_*i*_ = [*Cl* ^−^]_*o*_[*K* ^+^]_*o*_/[*K* ^+^]_*i*_ (i.e. *E*_*Cl*_ = *E*_*K*_) [74], even a small increase in [*K*]_*o*_ in the mature brain will reverse *Cl* ^−^ transport, from efflux to influx. [*K*]_*i*_, [*Cl*]_*i*_, and [*Cl*]_*o*_ are intracellular *K* ^+^, *Cl* ^−^, and extracellular *Cl* ^−^ concentrations respectively.

KCC2 in our model is formulated by a logarithmic function [26, 33]

$$\begin{array}{c} I_{KCC}=\rho_{KCC}log(\frac{{\left[K\right]}_{i}{\left[Cl\right]}_{i}}{{\left[K\right]}_{o}{\left[Cl\right]}_{o}}), \end{array}$$ (6)

where *ρ*_*KCC*_ is the maximum strength of KCC2 and estimated using the peak conductance given in [75]. In [33], we also included the *Na* ^+^/*K* ^+^/2*Cl* ^−^ (NKCC1) cotransporter, however, the results in this paper qualitatively remain the same without NKCC1 and hence it is excluded from the model. Nevertheless, NKCC1 should be included while modeling neurons from the embryonic or early postnatal brain [33, 74] and perhaps from pathological conditions where there may be aberrant transporter expression [76].

Intracellular *Na* ^+^ concentration is controlled by transient *Na* ^+^, *Na* ^+^ leak, and *Na* ^+^ − *K* ^+^ pump currents [15]

$$\begin{array}{c} \frac{d{\left[Na\right]}_{i}}{dt}=\frac{1}{\tau }\gamma (I_{Na}+I_{NaL}-3I_{pump}). \end{array}$$ (7)

Previously, we assumed that the flow of *Na* ^+^ into the cell is compensated by the flow of *K* ^+^ out of the cell so that [*K*]_*i*_ can be approximated by [15]

$$\begin{array}{c} {\left[K\right]}_{i}=140\text{mM}+\left(18\text{mM}-{\left[Na\right]}_{i}\right). \end{array}$$ (8)

We also assumed that the total amount of sodium is conserved, and hence only one differential equation for sodium is needed [15], so that

$$\begin{array}{c} {\left[Na\right]}_{o}=144\text{mM}-\beta \left({\left[Na\right]}_{i}-18\text{mM}\right), \end{array}$$ (9)

where 140mM, 18mM, and 144mM in the above equations are the normal resting values of [*K*]_*i*_, [*Na*]_*i*_, and [*Na*]_*o*_ respectively.

The intracellular *Cl* ^−^ concentration is modeled by *Cl* ^−^ leak and *K* ^+^ − *Cl* ^−^ cotransporter [33]

$$\begin{array}{c} \frac{d{\left[Cl\right]}_{i}}{dt}=\frac{1}{\tau }(\gamma I_{ClL}-\frac{I_{KCC}}{\beta }), \end{array}$$ (10)

and the extracellular *Cl* ^−^ concentration, [*Cl*]_*o*_, is set according to the conservation of charge due to *Na* ^+^, *K* ^+^, *Cl* ^−^, and *Ca* ^+^ ions in the extracellular space i.e.

$$\begin{array}{c} {\left[Cl\right]}_{o}={\left[K\right]}_{o}+{\left[Na\right]}_{o}+2.0{\left[Ca\right]}_{o}. \end{array}$$ (11)

Where [*Ca*]_*o*_ = 1mM is the extracellular calcium (*Ca* ^2+^) concentration.

The main conclusions in this paper qualitatively remain the same when [*K*]_*i*_, [*Na*]_*o*_, and [*Cl*]_*o*_ are modeled by rate equations governed by various fluxes (see “Results” section) instead of the simplified version due to the conservations shown above. This is consistent with our previous findings where we demonstrated that the unification of SZ and SD is qualitatively preserved when using simplified conservation equations [33]. However, the simplification due to conservations is helpful in performing bifurcation analysis using numerical solvers based on continuation methods such as XPPAUT [77]. Such simplifications are common and especially important in neuronal models where multiple time-scales are involved as in our model [15, 33, 78].

The reversal potentials for *K* ^+^, *Na* ^+^ and *Cl* ^−^ are updated based on the instantaneous ion concentrations using the Nernst equations

$$\begin{array}{ccc} V_{k} & = & 26.64\;\text{ln}(\frac{{\left[K\right]}_{o}}{{\left[K\right]}_{i}}),\\ V_{Na} & = & 26.64\;\text{ln}(\frac{{\left[Na\right]}_{o}}{{\left[Na\right]}_{i}}),\\ V_{Cl} & = & 26.64\;\text{ln}(\frac{{\left[Cl\right]}_{i}}{{\left[Cl\right]}_{o}}). \end{array}$$ (12)

The dynamic ion concentrations feedback into the Hodgkin-Huxley type Eqs (1, 2) through Eq (12). A more complex formulation would employ Goldman-Hodgkin-Katz voltage and current equations, which were found qualitatively similar in terms of unification in recent work [33].

### Dynamic cell volume

Intense neuronal firing during SZ, SD, AD and the relevant changes in ion concentrations cause cell swelling. We use the formalism of [14] to model the change in cell volume

$$\begin{array}{c} \hat{Vol}=Vol_{initial}\times \left(1.1029-0.1029\times exp\left(\left(\pi_{o}-\pi_{i}\right)/20\right)\right), \end{array}$$ (13)

where $\hat{Vol}$ and *Vol*_*initial*_ are the instantaneous and initial volumes of the cell. *π*_*o*_ and *π*_*i*_ are the sums of all ion concentrations outside and inside the cell respectively, i.e.

$$\begin{array}{ccc} \pi_{i} & = & {\left[Na\right]}_{i}+{\left[Cl\right]}_{i}+{\left[K\right]}_{i}+{\left[A\right]}_{i}+{\left[Ca\right]}_{i},\\ \pi_{o} & = & {\left[Na\right]}_{o}+{\left[Cl\right]}_{o}+{\left[K\right]}_{o}+{\left[A\right]}_{o}+{\left[Ca\right]}_{o}. \end{array}$$

[*A*]_*i*_ = 132.1*mM* [79] and [*A*]_*o*_ = 18*mM* are the intra- and extracellular concentrations of the impermeable anions. The value of [*A*]_*o*_ is based on charge balance under resting conditions using [*Cl*]_*o*_ = 132mM, and [*Ca*]_*i*_ = 100*nM* is the intracellular *Ca* ^2+^ concentration. Following [79], we implement the change in the cell volume as a first-order process.

$$\begin{array}{c} \frac{dVol}{dt}=\frac{1}{\tau_{v}}(\hat{Vol}-Vol) \end{array}$$ (14)

The time-scale of volume change, *τ*_*v*_, is important for the spontaneous SZ–SD transition. For the transition to happen, the recovery of the cell from the swollen state would have to be slower than the electrical response changes during a SZ. The cell would also have to be in a SZ state for long enough to produce this effect, and that lower levels of [*K*]_*o*_, with shorter duration seizures, would keep the cell out of SD. If the cell were to swell and shrink very fast so that it would recover its pre-seizure volume before going into next seizure, the cell would only exhibit spontaneous periodic seizures without going through the SZ–SD transition. To ensure the robustness of our results and that the spontaneous SZ–SD transition seen in our model occurs even with faster volume changes, we considered *τ*_*v*_ = 50ms, significantly smaller than the previously used phenomenological value of 250ms [33, 79]. Nevertheless, we tested a wide range of *τ*_*v*_ values and found that the model exhibits spontaneous SZ–SD transition for 40ms ≤ *τ*_*v*_ ≤ 360ms. For *τ*_*v*_ < 40ms, the cell recovers from swelling before going into seizure for the second time. For *τ*_*v*_ > 360ms, cell still exhibits spontaneous SZ–SD transition, however, it seizes more than once before going into SD state due to the slower change in volume. The number of SZs before the cell transitions into SD increases as we increase *τ*_*v*_. For example, for *τ*_*v*_ = 400ms, 500ms, and 1000ms, the cell seizes twice, thrice, and seven times respectively before making a transition to SD. While, the spontaneous SZ–SD transition itself is a robust phenomenon, *τ*_*v*_ > 360ms causes the cell to seize multiple times prior to the transition. The rest of the results including all bifurcation diagrams in the paper qualitatively remain the same as we change *τ*_*v*_.

In simulations where intracellular volume is treated as variable, the conductance densities for *Na* ^+^, *K* ^+^, and *Cl* ^−^ currents (*g*_*Na*_ etc) are modified so that the total conductance for a channel type over the whole cell remains fixed even though the conductance per unit area is changing

$$\begin{array}{c} g_{X}={\bar{g}}_{X}(A_{ss}/A_{ins}). \end{array}$$ (15)

Where *A*_*ss*_ and *A*_*ins*_ are the steady state (initial) and instantaneous cell surface areas respectively. ${\bar{g}}_{X}$ and *g*_*X*_ are the conductance densities in case of fixed and changing volumes respectively. The effect of dynamic volume changes on membrane capacitance is negligible and not included. For example, the total capacitance of the cell during the spontaneous transitions between SZ and SD shown in Fig 2 increased from initial 2.9074pF to 3.0295pF as the cell swelled, a variation that did not change the results qualitatively.

The dynamic change in volume causes the concentration of a given ion specie to change for a fixed number of ions. Thus, the dynamic volume leads to an additional flux term in the ion concentration equations. Thus the rate equation for [*K*]_*o*_ (Eq 3) becomes

$$\begin{array}{c} \frac{d{\left[K\right]}_{o}}{dt}=\frac{1}{\tau }(\gamma \beta \left(I_{K}+I_{KL}-2I_{pump}\right)-I_{diff}-I_{glia}+I_{KCC})-(\frac{dVol_{o}}{dt})(\frac{{\left[K\right]}_{o}}{Vol_{o}}). \end{array}$$

Where *Vol*_*o*_ is the extracellular volume. Similarly, the rate equations for [*Na*]_*i*_ (Eq 7) and [*Cl*]_*i*_ (Eq 10) also have additional terms equal to $\left(\frac{-dVol}{dt}\right)\left(\frac{{\left[Na\right]}_{i}}{Vol_{i}}\right)$ and $\left(\frac{-dVol}{dt}\right)\left(\frac{{\left[Cl\right]}_{i}}{Vol_{i}}\right)$ respectively. However, these fluxes do not change the results presented in this paper qualitatively and are not considered.

Notice that there are four different time-scales in our model: fast (variables *V*, *n*, and *h*; Eq (1)), intermediate ([*K*]_*o*_, [*Na*]_*i*_; Eqs (3, 7)), slow ([*O*_2_], [*Cl*]_*i*_; Eqs (5, 10)), and infra-slow (*Vol*; Eq (14)). Although we focus on the implications of dynamic intracellular volume for cell behavior in pathological states in this study, we will also analyze the evolution of other slow variables during these states and how they effect cell pathology.

### Numerical methods

The coupled differential equations are solved in Fortran 90 using 4th order Runge-Kutta method. The steady states in Fig 1A, and the bifurcation diagrams in Figs 5 and 6 are generated using XPPAUT software [77]. In the bifurcation diagrams of [*K*]_*o*_ (and other slow variables), XPPAUT could only capture the small amplitude oscillations in [*K*]_*o*_ due to individual membrane potential spikes. It could not capture the high amplitude low frequency oscillations due to periodic SZ and SD events. Therefore, we simulated the periodic orbits in Figs 1A, 3, S1, and S2 Figs using Fortran 90. The codes reproducing key results in the paper are given in Supporting Information Text S1.

## Supporting Information

S1 Fig One-parameter bifurcation in the model when [*K*]_*i*_, [*Na*]_*o*_, and [*Cl*]_*i*_ are formulated by rate equations. That is, we replace Eqs (8, 9, and 11) by *d*[*K*]_*i*_/*dt* = (1/*τ*)(−*γ*(*I*_*K*_ + *I*_*KL*_ − 2.0*I*_*pump*_) − *I*_*KCC*_/*β*), *d*[*Na*]_*o*_/*dt* = (1/*τ*)(−*γβ*(*I*_*Na*_ + *I*_*NaL*_ − 3*I*_*pump*_), and *d*[*Cl*]_*o*_/*dt* = (1/*τ*)(−*γβI*_*ClL*_ − *I*_*KCC*_/*β*) respectively. We consider *Vol* (Eq 14) as a bifurcation parameter and simulate Eqs (1, 3, 5, 7, 10) together with the above three differential equations. The maximum and minimum of [*K*]_*o*_ as a function of *r*_*in*_ (left panel) shows that the model cell goes through the transition between SZ and SD qualitatively in the same manner as the model cell where [*K*]_*i*_, [*Na*]_*o*_, and [*Cl*]_*i*_ are formulated by conservation equations. The unstable steady state is not shown in the left panel. The right panels show SZ (top) and mixed SZ-SD (bottom) behaviors for *r*_*in*_ = 4.82*μm* and 4.87*μm* respectively.
(TIFF)

S2 Fig The transition between SZ and SD is mainly caused by the change in *β*. (A) Bifurcation diagram for the model as a function of *r*_*in*_ at fixed *β* = 7.68 (black) and 10.5 (blue). (B) Maxima and minima of [*K*]_*o*_ oscillations as a function of *β* at fixed *r*_*in*_ = 4.65*μm* (black) and 4.8*μm* (blue). Bullets and red lines represent stable periodic orbit and steady states respectively. The unstable steady states are not shown.
(TIFF)

S3 Fig Dynamic *Cl* ^−^ concentrations and diminished *K* ^+^ diffusion between extracellular space and blood vessels is necessary for AD. Membrane potential of the cell in response to ED with fixed [*Cl*]_*i*_ = 8*mM* and [*Cl*]_*o*_ = 140*mM* (A) and normal *K* ^+^ diffusion between blood vessels and extracellular space (B). All other equations and parameters are the same as in Fig 7A (black line) that can be used for comparison.
(TIFF)

S1 Codes The codes reproducing the main results in the paper. (ZIP)

## References

[1] Somjen GG (2004) Ions in the brain. New York: Oxford University Press.

[2] Andrew RD, MacVicar B (1994) Imaging cell volume changes and neuronal excitation in the hippocampal slice. Neuroscience 62: 371–383. 10.1016/0306-4522(94)90372-7 7830884

[3] Fields RD (2011) Signaling by neuronal swelling. Science Signaling 4: tr1 10.1126/scisignal.4155tr1 21224445 PMC3201844

[4] Roper SN, Obenaus A, Dudek FE (1992) Osmolality and nonsynaptic epileptiform bursts in rat ca1 and dentate gyrus. Annals of Neurology 31: 81–85. 10.1002/ana.410310115 1543352

[5] Andrew RD (1991) Seizure and acute osmotic change: clinical and neurophysiological aspects. Journal of the Neurological Sciences 101: 7–18. 10.1016/0022-510X(91)90013-W 2027029

[6] Snow RW, Dudek FE (1984) Electrical fields directly contribute to action potential synchronization during convulsant-induced epileptiform bursts. Brain Research 323: 114–118. 10.1016/0006-8993(84)90271-3 6525502

[7] Rosen AS, Andrew RD (1990) Osmotic effects upon excitability in rat neocortical slices. Neuroscience 38: 579–590. 10.1016/0306-4522(90)90052-6 2270133

[8] Hill B, Schubert ED, Nokes MA, Michelson RP (1977) Laser interferometer measurement of changes in crayfish axon diameter concurrent with action potential. Science 196: 426–428. 10.1126/science.850785 850785

[9] Iwasa K, Tasaki I, Gibbons RC (1980) Swelling of nerve fibers associated with action potentials. Science 210: 338–339. 10.1126/science.7423196 7423196

[10] Bikson M, Hahn PJ, Fox JE, Jefferys JGR (2003) Depolarization block of neurons during maintenance of electrographic seizures. J Neurophysiol 90: 2402–2408. 10.1152/jn.00467.2003 12801897

[11] Lauritzen M (1987) Cortical spreading depression as a putative migraine mechanism. Trends in Neurosciences 10: 8–13. 10.1016/0166-2236(87)90115-9

[12] Bureš J, Burešová O (1981) Cerebral [*K* ^+^]*e* increase as an index of the differential susceptibility of brain structures to terminal anoxia and electroconvulsive shock. Journal of Neurobiology 12: 211–220. 10.1002/neu.480120303 7276923

[13] Kager H, Wadman W, Somjen G (2002) Conditions for the triggering of spreading depression studied with computer simulations. J Neurophysiol 88: 2700–2712. 10.1152/jn.00237.2002 12424305

[14] Kager H, Wadman W, Somjen G (2007) Seizure-like afterdischarges simulated in a model neuron. J Comput Neurosci 22: 105–128. 10.1007/s10827-006-0001-y 17053996

[15] Cressman JR, Ullah G, Žiburkus J, Schiff SJ, Barreto E (2009) The influence of sodium and potassium dynamics on excitability, seizures, and the stability of persistent states: I. Single neuron dynamics. J Comput Neurosci 26: 159–70. 10.1007/s10827-008-0132-4 19169801 PMC2704057

[16] Krishnan GP, Bazhenov M (2011) Ionic dynamics mediate spontaneous termination of seizures and postictal depression state. The Journal of Neuroscience 31: 8870–82. 10.1523/JNEUROSCI.6200-10.2011 21677171 PMC3163257

[17] Ingram JM, Zhang C, Xu J, Schiff SJ (2013) FRET excited ratiometric oxygen sensing in living tissue. Journal of Neuroscience Methods 214: 45–51. 10.1016/j.jneumeth.2013.01.002 23333398 PMC3664065

[18] Wei Y, Ullah G, Ingram J, Schiff SJ (2014) Oxygen and seizure dynamics: II. computational modeling. Journal of Neurophysiology 112: 213–223. 10.1152/jn.00541.2013 24671540 PMC4064403

[19] Kager H, Wadman WJ, Somjen GG (2000) Simulated seizures and spreading depression in a neuron model incorporating interstitial space and ion concentrations. Journal of Neurophysiology 84: 495–512. 10899222 10.1152/jn.2000.84.1.495

[20] Brisson C, Andrew RD (2012) A neuronal population in hypothalamus that dramatically resists acute ischemic injury compared to neocortex. Journal of Neurophysiology 108: 419–430. 10.1152/jn.00090.2012 22514289

[21] Brisson CD, Lukewich MK, Andrew RD (2013) A distinct boundary between the higher brain’s susceptibility to ischemia and the lower brain’s resistance. PLoS One 8: e79589 10.1371/journal.pone.0079589 24223181 PMC3819273

[22] Andrew RD, Labron MW, Boehnke SE, Carnduff L, Kirov SA (2007) Physiological evidence that pyramidal neurons lack functional water channels. Cerebral Cortex 17: 787–802. 10.1093/cercor/bhk032 16723408

[23] Zeuthen T (2010) Water-transporting proteins. Journal of Membrane Biology 234: 57–73. 10.1007/s00232-009-9216-y 20091162

[24] Hoffmann EK, Lambert IH, Pedersen SF (2009) Physiology of cell volume regulation in vertebrates. Physiological Reviews 89: 193–277. 10.1152/physrev.00037.2007 19126758

[25] Risher WC, Andrew RD, Kirov SA (2009) Real-time passive volume responses of astrocytes to acute osmotic and ischemic stress in cortical slices and in vivo revealed by two-photon microscopy. Glia 57: 207–221. 10.1002/glia.20747 18720409 PMC2635108

[26] Østby I, Øyehaug L, Einevoll GT, Nagelhus EA, Plahte E, et al (2009) Astrocytic mechanisms explaining neural-activity-induced shrinkage of extraneuronal space. PLoS ComputationalBiology 5: e1000272. 10.1371/journal.pcbi.1000272 PMC2613522 19165313

[27] Walz W (1991) Accumulation of intracellular bicarbonate accounts for the missing anion during potassium-evoked swelling of cortical type-1-like astrocytesa. Annals of the New York Academy of Sciences 633: 589–591. 10.1111/j.1749-6632.1991.tb15671.x 1789587

[28] Walz W, Hinks EC (1985) Carrier-mediated kcl accumulation accompanied by water movements is involved in the control of physiological k^+^ levels by astrocytes. Brain Research 343: 44–51. 10.1016/0006-8993(85)91156-4 4041856

[29] Leão A (1944) Spreading depression of activity in the cerebral cortex. J Neurophysiol 7: 359–390. 10.1152/jn.1947.10.6.409 20268874

[30] Traynelis SF, Dingledine R (1988) Potassium-induced spontaneous electrographic seizures in the rat hippocampal slice. Journal of Neurophysiology 59: 259–276. 3343603 10.1152/jn.1988.59.1.259

[31] Žiburkus J, Cressman JR, Barreto E, Schiff SJ (2006) Interneuron and pyramidal cell interplay during in vitro seizure-like events. Journal of Neurophysiology 95: 3948–54. 10.1152/jn.01378.2005 16554499 PMC1469233

[32] Czéh G, Aitken PG, Somjen GG (1993) Membrane currents in CA1 pyramidal cells during spreading depression (SD) and SD-like hypoxic depolarization. Brain Research 632: 195–208. 10.1016/0006-8993(93)91154-K 8149228

[33] Wei Y, Ullah G, Schiff SJ (2014) Unification of neuronal spikes, seizures, and spreading depression. The Journal of Neuroscience 34: 11733–11743. 10.1523/JNEUROSCI.0516-14.2014 25164668 PMC4145176

[34] Hübel N, Dahlem MA (2014) Dynamics from seconds to hours in Hodgkin–Huxley model with time–dependent ion concentrations and buffer reservoirs. arXiv:14043031. 10.1371/journal.pcbi.1003941 PMC4256015 25474648

[35] Annunziato L (2009) New strategies in stroke intervention: ionic transporters, pumps, and new channels, volume 7626 Springer.

[36] Ziburkus J, Cressman JR Jr, Barreto E, Schiff SJ (2006) Interneuron and pyramidal cell interplay during in vitro seizure-like events. J Neurophysiol 95: 3948–3954. 10.1152/jn.01378.2005 16554499 PMC1469233

[37] Anderson TR, Andrew RD (2002) Spreading depression: imaging and blockade in the rat neocortical brain slice. Journal of Neurophysiology 88: 2713–2725. 10.1152/jn.00321.2002 12424306

[38] Haglund M, Schwartzkroin P (1999) Role of Na-K pump potassium regulation and IPSPs in seizures and spreading depression in immature rabbit hippocampal slices. J Neurophysiol 63. 10.1152/jn.1990.63.2.225 2313342

[39] Zandt BJ, Stigen T, ten Haken B, Netoff T, van Putten MJ (2013) Single neuron dynamics during experimentally induced anoxic depolarization. Journal of Neurophysiology 110: 1469–1475. 10.1152/jn.00250.2013 23825394

[40] Dahlem MA, Graf R, Strong AJ, Dreier JP, Dahlem YA, et al (2010) Two-dimensional wave patterns of spreading depolarization: retracting, re-entrant, and stationary waves. Physica D: Nonlinear Phenomena 239: 889–903. 10.1016/j.physd.2009.08.009

[41] Rogawski MA (2010) Migraine and epilepsy: Shared mechanisms? Epilepsia 51: 80 10.1111/j.1528-1167.2010.02866.x

[42] Somjen GG (2001) Mechanism of spreading depression and hypoxic spreading depression-like depolarization. Physiological Reviews 81: 1065–1096. 11427692 10.1152/physrev.2001.81.3.1065

[43] Neishtadt AI (1988) Prolongation of the loss of stability in the case of dynamic bifurcations. ii. Differential Equations 24: 171–176.

[44] Baer SM, Erneux T, Rinzel J (1989) The slow passage through a Hopf bifurcation: delay, memory effects, and resonance. SIAM Journal on Applied mathematics 49: 55–71. 10.1137/0149003

[45] Gebhardt C, Körner R, Heinemann U (2002) Delayed anoxic depolarizations in hippocampal neurons of mice lacking the excitatory amino acid carrier 1. Journal of Cerebral Blood Flow & Metabolism 22: 569–575. 10.1097/00004647-200205000-00008 11973429

[46] Schwartz-Bloom RD, Sah R (2001) *γ*-aminobutyric acida neurotransmission and cerebral ischemia. Journal of Neurochemistry 77: 353–371. 10.1046/j.1471-4159.2001.00274.x 11299298

[47] Kinney JP, Spacek J, Bartol TM, Bajaj CL, Harris KM, et al (2013) Extracellular sheets and tunnels modulate glutamate diffusion in hippocampal neuropil. Journal of Comparative Neurology 521: 448–464. 10.1002/cne.23181 22740128 PMC3540825

[48] Zandt BJ, ten Haken B, van Dijk J, van Putten MJ (2011) Neural dynamics during anoxia and the “wave of death”. PLoS One 6: e22127 10.1371/journal.pone.0022127 21779384 PMC3135620

[49] Arumugam TV, Okun E, Mattson MP (2010) Basis of Ionic Dysregulation in Cerebral Ischemia In: New Strategies in Stroke Intervention, Totowa, NJ: Humana Press, chapter 1. pp. 1–11.

[50] Hibino H, Inanobe A, Furutani K, Murakami S, Findlay I, et al (2010) Inwardly rectifying potassium channels: their structure, function, and physiological roles. Physiological Reviews 90: 291–366. 10.1152/physrev.00021.2009 20086079

[51] Witthoft A, Filosa JA, Karniadakis GE (2013) Potassium buffering in the neurovascular unit: Models and sensitivity analysis. Biophysical Journal 105: 2046–2054. 10.1016/j.bpj.2013.09.012 24209849 PMC3824545

[52] Gloor SM (1997) Relevance of na, k-atpase to local extracellular potassium homeostasis and modulation of synaptic transmission. FEBS Letters 412: 1–4. 10.1016/S0014-5793(97)00774-6 9257678

[53] Saly V, Andrew RD (1993) CA3 neuron excitation and epileptiform discharge are sensitive to osmolality. Journal of Neurophysiology 69: 2200–2208. 8350139 10.1152/jn.1993.69.6.2200

[54] Rosen AS, Andrew RD (1991) Glucose concentration inversely alters neocortical slice excitability through an osmotic effect. Brain Research 555: 58–64. 10.1016/0006-8993(91)90859-T 1933330

[55] Andrew RD, Fagan M, Ballyk BA, Rosen AS (1989) Seizure susceptibility and the osmotic state. Brain Research 498: 175–180. 10.1016/0006-8993(89)90417-4 2790471

[56] Azouz R, Alroy G, Yaari Y (1997) Modulation of endogenous firing patterns by osmolarity in rat hippocampal neurones. The Journal of Physiology 502: 175–187. 10.1111/j.1469-7793.1997.175bl.x 9234205 PMC1159580

[57] Oberheim NA, Tian GF, Han X, Peng W, Takano T, et al (2008) Loss of astrocytic domain organization in the epileptic brain. The Journal of Neuroscience 28: 3264–3276. 10.1523/JNEUROSCI.4980-07.2008 18367594 PMC6670598

[58] Devinsky O (2008) Epilepsy: Patient and family guide. Demos Medical Publishing.

[59] Foley J, Nguyen H, Bennett C, Muschol M (2010) Potassium accumulation as dynamic modulator of neurohypophysial excitability. Neuroscience 169: 65–73. 10.1016/j.neuroscience.2010.04.049 20433904

[60] Wechselberger M (2007) Canards. Scholarpedia 2: 1356 10.4249/scholarpedia.1356

[61] Lennie P (2003) The cost of cortical computation. Current Biology 13: 493–497. 10.1016/S0960-9822(03)00135-0 12646132

[62] Cressman JR, Ullah G, Ziburkus J, Schiff SJ, Barreto E (2011) Erratum to: The influence of sodium and potassium dynamics on excitability, seizures, and the stability of persistent states: I. single neuron dynamics. Journal of Computational Neuroscience 30: 781–781. 10.1007/s10827-008-0132-4 PMC2704057 19169801

[63] Hodgkin AL, Huxely A (1952) A quantitative description of membrane current and its application to conduction and excitation in nerve. J Physiol 117: 500–544. 10.1113/jphysiol.1952.sp004764 12991237 PMC1392413

[64] Gutkin BS, Laing CR, Colby CL, Chow CC, Ermentrout GB (2001) Turning on and off with excitation: the role of spike-timing asynchrony and synchrony in sustained neural activity. Journal of Computational Neuroscience 11: 121–134. 11717529 10.1023/a:1012837415096

[65] Rinzel J (1985) Excitation dynamics: insights from simplified membrane models. In: Fed. Proc. volume 44, pp. 2944–2946. 2415401

[66] Ullah G, Cressman JR Jr, Barreto E, Schiff SJ (2009) The influence of sodium and potassium dynamics on excitability, seizures, and the stability of persistent states: II. Network and glial dynamics. J Comput Neurosci 26: 171–183. 10.1007/s10827-008-0130-6 19083088 PMC2951284

[67] Ullah G, Schiff SJ (2009) Tracking and control of neuronal Hodgkin-Huxley dynamics. Phys Rev E 79: 040901 10.1103/PhysRevE.79.040901 PMC2713719 19518166

[68] Ullah G, Schiff SJ (2010) Assimilating seizure dynamics. PLoS Computational Biology 6: e1000776 10.1371/journal.pcbi.1000776 20463875 PMC2865517

[69] Fisher RS, Pedley TA, Prince DA (1976) Kinetics of potassium movement in norman cortex. Brain Res 101: 223–237. 10.1016/0006-8993(76)90265-1 1244970

[70] Scharrer E (1944) The blood vessels of the nervous tissue. Quart Rev Biol 19: 308–318. 10.1086/394698

[71] Paulson OB, Newman EA (1987) Does the release of potassium from astrocyte endfeet regulate cerebral blood flow? Science 237: 896–898. 10.1126/science.3616619 3616619 PMC2505270

[72] Kuschinsky W, Wahl M, Bosse O, Thurau K (1972) The dependency of the pial arterial and arteriolar resistance on the perivascular H+ and K+ conconcentrations. a micropuncture study. Eur Neurol 6: 92–95. 10.1159/000114473 5153461

[73] McCulloch J, Edvinsson L, Watt P (1982) Comparison of the effects of potassium and ph on the calibre of cerebral veins and arteries. Pflugers Arch 393: 95–98. 10.1007/BF00582399 7088689

[74] Payne JA, Rivera C, Voipio J, Kaila K (2003) Cation–chloride co-transporters in neuronal communication, development and trauma. Trends in Neurosciences 26: 199–206. 10.1016/S0166-2236(03)00068-7 12689771

[75] Lauf PK, Adragna NC (2001) K-Cl cotransport: properties and molecular mechanism. Cellular Physiology and Biochemistry 10: 341–354. 10.1159/000016357 11125215

[76] Cohen I, Navarro V, Clemenceau S, Baulac M, Miles R (2002) On the origin of interictal activity in human temporal lobe epilepsy in vitro. Science 298: 1418–1421. 10.1126/science.1076510 12434059

[77] Ermentrout G (2002) Simulating, analyzing, and animating dynamical systems: a guide to XPPAUT for researchers and students. Philadelphia, PA: Society for Industrial & Applied Mathematics.

[78] Rubin JE, Terman D (2004) High frequency stimulation of the subthalamic nucleus eliminates pathological thalamic rhythmicity in a computational model. Journal of Computational Neuroscience 16: 211–235. 15114047 10.1023/B:JCNS.0000025686.47117.67

[79] Kager H, Wadman WJ, Somjen GG (2007) Seizure-like afterdischarges simulated in a model neuron. J Comput Neurosci 22: 105–128. 10.1007/s10827-006-0001-y 17053996

