# Supplementary figures and images for: The Role of Cell Volume in the Dynamics of Seizure, Spreading Depression, and Anoxic Depolarization
Source: PLoS Comput Biol. 2015 Aug 14;11(8):e1004414. doi: 10.1371/journal.pcbi.1004414 (PMC4537206; doi:10.1371/journal.pcbi.1004414)

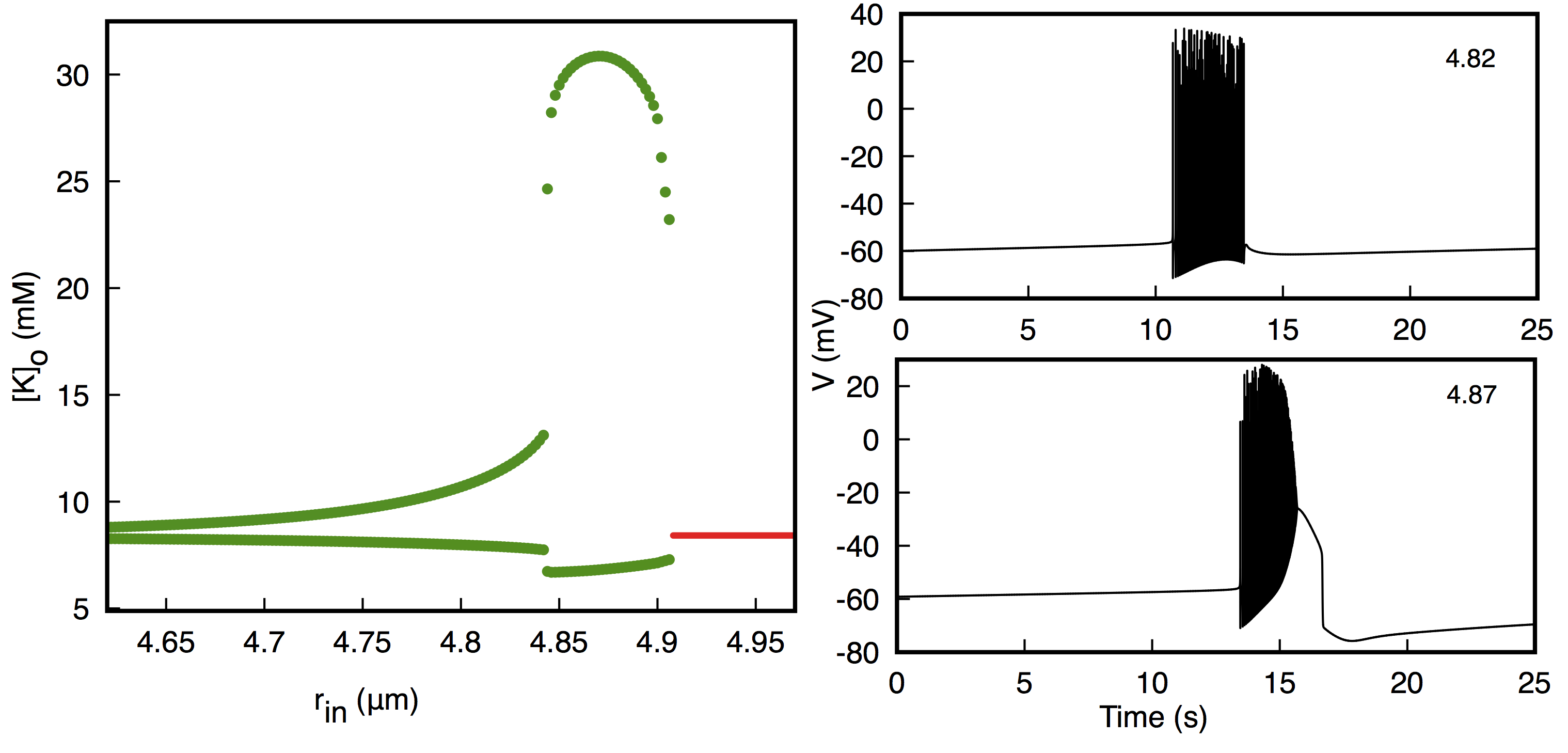

Supplement: S1 Fig — That is, we replace Eqs (8, 9, and 11) by d[K]i/dt = (1/τ)(−γ(I K + I KL − 2.0I pump) − I KCC/β), d[Na]o/dt = (1/τ)(−γβ(I Na + I NaL − 3I pump), and d[Cl]o/dt = (1/τ)(−γβI ClL − I KCC/β) respectively. We consider Vol (Eq 14) as a bifurcation parameter and simulate Eqs (1, 3, 5, 7, 10) together with the above three differential equations. The maximum and minimum of [K]o as a function of r in (left panel) shows that the model cell goes through the transition between SZ and SD qualitatively in the same manner as the model cell where [K]i, [Na]o, and [Cl]i are formulated by conservation equations. The unstable steady state is not shown in the left panel. The right panels show SZ (top) and mixed SZ-SD (bottom) behaviors for r in = 4.82μm and 4.87μm respectively. (TIFF) [file pcbi.1004414.s001.tiff]

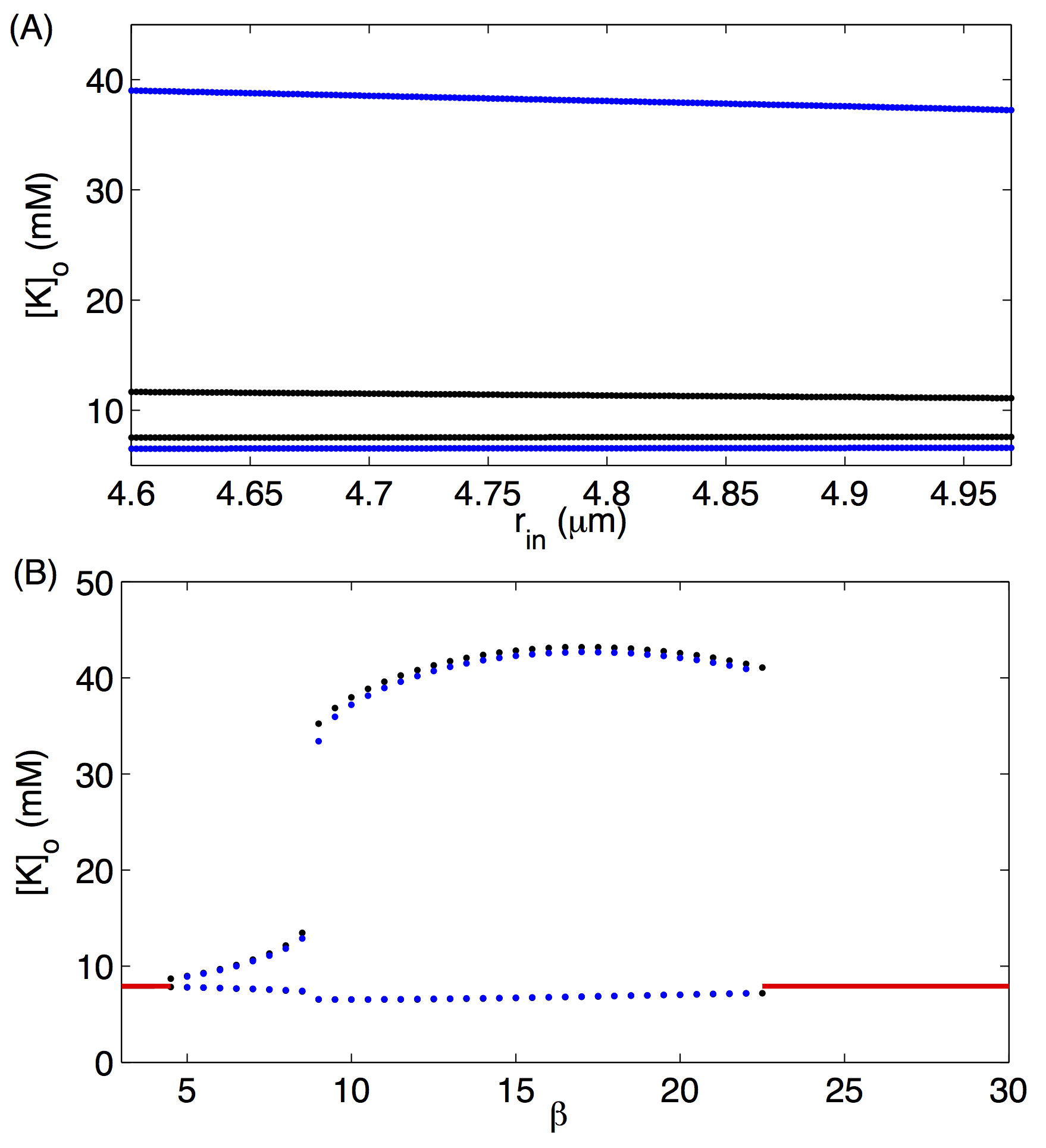

Supplement: S2 Fig — (A) Bifurcation diagram for the model as a function of r in at fixed β = 7.68 (black) and 10.5 (blue). (B) Maxima and minima of [K]o oscillations as a function of β at fixed r in = 4.65μm (black) and 4.8μm (blue). Bullets and red lines represent stable periodic orbit and steady states respectively. The unstable steady states are not shown. (TIFF) [file pcbi.1004414.s002.tiff]

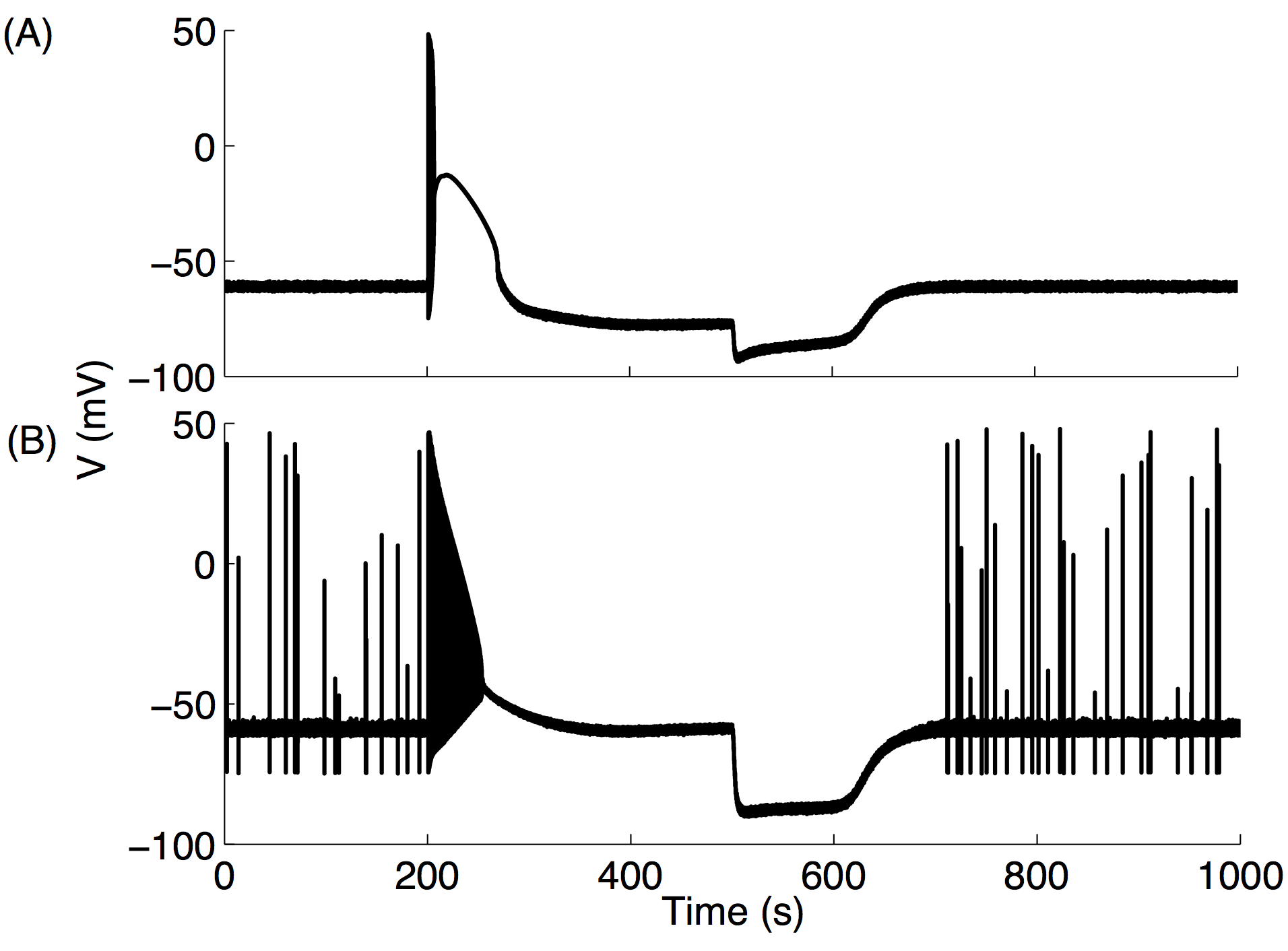

Supplement: S3 Fig — Membrane potential of the cell in response to ED with fixed [Cl]i = 8mM and [Cl]o = 140mM (A) and normal K + diffusion between blood vessels and extracellular space (B). All other equations and parameters are the same as in Fig 7A (black line) that can be used for comparison. (TIFF) [file pcbi.1004414.s003.tiff]
